# Economic evaluation of prostate cancer risk assessment methods: A cost‐effectiveness analysis using population data

**DOI:** 10.1002/cam4.6587

**Published:** 2023-09-23

**Authors:** Tima Mohammadi, Daphne P. Guh, Alexander C. T. Tam, Reka E. Pataky, Peter C. Black, Alan So, Larry D. Lynd, Wei Zhang, Annalijn I. Conklin

**Affiliations:** ^1^ Centre for Advancing Health Outcomes (formerly Centre for Health Evaluation and Outcome Sciences), Providence Health Care Research Institute St. Paul's Hospital Vancouver British Columbia Canada; ^2^ Canadian Centre for Applied Research in Cancer Control, BC Cancer Vancouver British Columbia Canada; ^3^ Department of Urologic Sciences, Faculty of Medicine University of British Columbia Vancouver British Columbia Canada; ^4^ Faculty of Pharmaceutical Sciences University of British Columbia Vancouver British Columbia Canada

**Keywords:** economic evaluation, health administrative data, prostate cancer, screening

## Abstract

**Background:**

The current prostate cancer (PCa) screening standard of care (SOC) leads to unnecessary biopsies and overtreatment because decisions are guided by prostate‐specific antigen (PSA) levels, which have low specificity in the gray zone (3–10 ng/mL). New risk assessment tools (RATs) aim to improve biopsy decision‐making. We constructed a modeling framework to assess new RATs in men with gray zone PSA from the British Columbia healthcare system's perspective.

**Methods:**

We evaluated the cost‐effectiveness of a new RAT used in biopsy‐naïve men aged 50+ with a PSA of 3–10 ng/mL using a time‐dependent state‐transition model. The model was informed by engaging patient partners and using linked administrative health data, supplemented with published literature. The incremental cost‐effectiveness ratio and the probability of the RAT being cost‐effective were calculated. Probabilistic analysis was used to assess parameter uncertainty.

**Results:**

In the base case, a RAT based on an existing biomarker's characteristics was a dominant strategy associated with a cost savings of $44 and a quality‐adjusted life years (QALY) gain of 0.00253 over 18 years of follow‐up. At a cost‐effectiveness threshold of $50,000/QALY, the probability that using a RAT is cost‐effective relative to the SOC was 73%. Outcomes were sensitive to RAT costs and accuracy, especially the detection rate of high‐grade PCa. Results were also impacted by PCa prevalence and assumptions about undetected PCa survival.

**Conclusions:**

Our findings showed that a more accurate RAT to guide biopsy can be cost‐effective. Our proposed general model can be used to analyze the cost‐effectiveness of any novel RAT.

## 1 BACKGROUND

Prostate cancer (PCa) is the most common cancer in men in many countries, including Canada.^1^ Most cases are diagnosed by needle biopsy triggered by an elevated serum prostate‐specific antigen (PSA) level. PSA screening has been shown to improve outcomes and reduce PCa‐specific mortality.^2^ However, PSA is an organ‐specific and not a cancer‐specific marker,^3^ and increased levels can be observed in other health states.^4^ When used alone, PSA as a cancer screening tool is controversial, given that there is a lack of an optimal cutoff value that can provide both high sensitivity and high specificity.^5^, ^6^ There is a ‘gray zone’ of PSA values between 3.0 and 10.0 ng/mL where the test performs especially poorly: Barry^7^ estimated that 75% of men with gray zone PSA do not have PCa on prostate biopsy. Biopsies are invasive and carry risks of bleeding, infection,^8^, ^9^, ^10^ and anxiety in patients.^9^ Overall, the limited specificity of PSA for the detection of clinically significant PCa is a source of unnecessary biopsies,^11^, ^12^, ^13^ which can lead to overtreatment, compromising patient safety and quality of care.^14^, ^15^ Therefore, newer more specific and sensitive tools are needed to assess the risk of PCa before biopsy, especially for men with PSA levels within the gray zone.

A number of risk assessment tools (RATs) have been proposed to facilitate the decision‐making process to determine if men with elevated PSA require a biopsy. These tools include PSA kinetics, PSA density, percent‐free PSA, urine or liquid biomarker panels, and prostate risk calculators.^1^ Additionally, the role of magnetic resonance imaging (MRI) in improving the diagnosis of PCa in biopsy‐naïve men has been evaluated.^16^, ^17^, ^18^ A key component of healthcare and policy decision‐making of a new health technology is an economic evaluation to assess the costs against the benefits of the new technology compared to the standard of care (SOC). Previous studies have compared the cost and effectiveness of specific biomarkers to strategies using PSA alone,^19^, ^20^, ^21^ and some have evaluated the value of using MRI to guide biopsy.^22^, ^23^, ^24^, ^25^ Reviewing these studies showed that modeling the cost and effectiveness of PCa RATs and/or diagnostic tools involves considerable complexity and variation.^26^ As such, this economic evaluation study aimed to develop and implement a general framework for cost‐effectiveness analysis of RATs as an additional tool to aid decision‐making in PCa diagnosis among men with PSA within the gray zone. We used a decision analytical modeling approach to fully consider uncertainties and accurately measure the impact of RATs on health‐related quality of life, mortality, and real costs using province‐wide health administrative data.

## 2 METHODS

### 2.1 Model structure

We developed a time‐dependent cohort state‐transition model, a dynamic model in which a hypothetical cohort of individuals transitions between different health states and the transition probabilities change over time. This model was used to evaluate the cost and effectiveness of a RAT strategy following the PSA compared with SOC (PSA alone) to guide the initial biopsy decision. The analysis was from the perspective of the publicly funded healthcare payer in British Columbia (BC), Canada. The target population consisted of men aged ≥50 years,^27^ with a PSA of 3–10 ng/mL. The main diagnostic and PCa treatment pathway was similar to the patient pathway modeled in previous studies.^19^, ^20^, ^28^ In defining our model structure and assumptions, however, we also sought feedback from five patient partners and three clinicians, two from BC and one from Ontario. The significant variation in treatment pathways was identified as an important consideration in our stakeholder engagement process. This confirmed the need for BC‐specific data to inform costs and treatment distributions and for modifying the treatment pathway to reflect local PCa care.

Figure S1 shows the pathways in the model structure for the first year. A cohort of our target population has a probability of going through one pathway within the first year and then entering a corresponding time‐dependent state‐transition model. The model consists of different health states: (1) treatment options within the first year of diagnosis and post‐recovery period (radical prostatectomy, radiotherapy, androgen deprivation therapy (ADT), and/or chemo); (2) watchful waiting or active surveillance (WW/AS); and (3) undetected high‐grade (Gleason score ≥7) and low‐grade (Gleason score ≤6) patients.

The cycle length was 1 year. The cohort of patients in both strategies was simulated over a time horizon of 18 years similar to cost‐effectiveness studies^19^, ^20^, ^28^ based on the median follow‐up time of survival data for patients with PCa from the Bill‐Axelson et al. study.^29^ We assumed that in the SOC strategy, all men with a PSA in the gray zone undergo a transrectal ultrasound‐guided biopsy. The PCa diagnosis is based on the results of the biopsy. In the RAT strategy, a tool (e.g., biomarker) is used to evaluate the risk of PCa in individuals with PSA levels between 3 and 10 ng/mL. Biopsies are performed only among men whose results indicate a high risk of having PCa. It is assumed that undetected PCa (false negatives of the RAT) might be detected subsequently by clinical symptoms. Therefore, we made corresponding assumptions for costs, mortality, and disutility for this group, which are described below.

### 2.2 Model inputs

We used administrative health data to inform most of the model parameters,^30^, ^31^, ^32^, ^33^, ^34^, ^35^, ^36^ supplemented with data from a comprehensive literature review. The point estimates and assigned distributions for the model parameters are presented in Tables 1 and 2, and Table S1.

**TABLE 1:** Model probability parameters, point estimates, probability distribution, and data sources used in the cost‐effectiveness model.

Parameter	Point estimate	Probability distribution	References
Probability of PCa in gray zone	0.25	Fixed	American Cancer Society^56^
Probability of high‐grade in case of PCa	0.727	Beta (7204, 2707)	BC data
Probability of low‐grade in case of PCa	0.273	Beta (2707, 7204)	BC data
Ratio of advanced to early stage in all HG‐PCa	0.290	Fixed	BC data
Ratio of advanced to early stage in all LG‐PCa	0.050	Fixed	BC data
Ratio of advanced to early stage in WW/AS (untreated) HG‐PCa	0.052	Fixed	BC data
Ratio of advanced to early stage in WW/AS (untreated) LG‐PCa	0.007	Fixed	BC data
Sensitivity of RAT for HG‐PCa	0.957	Fixed	19, 20, 41
Specificity of RAT for LG‐PCa	0.336	Fixed	19, 20, 41
Specificity of RAT for No‐PCa	0.608	Fixed	19, 20, 41
Treatment distribution			
Probability of radical prostatectomy with HG‐PCa	0.449	Beta (3231, 3973)	BC data
Probability of radiotherapy with HG‐PCa	0.376	Beta (2712, 4492)	BC data
Probability of ADT and/or chemo with HG‐PCa	0.060	Beta (435, 6769)	BC data
Probability of WW/AS (untreated) with HG‐PCa	0.115	Beta (826, 6378)	BC data
Probability of radical prostatectomy with LG‐PCa	0.238	Beta (643, 2064)	BC data
Probability of radiotherapy with LG‐PCa	0.095	Beta (256, 2451)	BC data
Probability of ADT and/or chemo with LG‐PCa	0.013	Beta (36, 2671)	BC data
Probability of WW/AS (untreated) with LG‐PCa	0.655	Beta (1772, 935)	BC data
PCa‐specific mortality			
Log of PCa‐specific death rate‐radical prostatectomy with HG‐PCa	−5.65997	Normal (−5.65997, 0.1222)	BC data
Log of PCa‐specific death rate‐radiotherapy with HG‐PCa	−4.68996	Normal (−4.68996, 0.0822)	BC data
Log of PCa‐specific death rate‐ADT and/or chemo with HG‐PCa	−3.07944	Normal (−3.07944, 0.1010)	BC data
Log of PCa‐specific death rate‐WW/AS (untreated) with HG‐PCa	−4.82612	Normal (−4.82612, 0.1644)	BC data
Log of PCa‐specific death rate‐undetected HG‐PCa	−4.82612	Normal (−4.82612, 0.1644)	BC data and assumption
Log of PCa‐specific death rate‐any treatment with HG‐PCa	−4.78533	Normal (−4.78533, 0.0565)	BC data
Hazard ratio for death (treated vs. undetected) with HG‐PCa	0.8700	Lognormal (−0.196, 0.336)	^29^
Log of PCa‐specific death rate‐radical prostatectomy with LG‐PCa	−7.25097	Normal (−7.25097, 0.5774)	BC data
Log of PCa‐specific death rate‐radiotherapy with LG‐PCa	−4.96807	Normal (−4.96807, 0.2887)	BC data
Log of PCa‐specific death rate‐ADT and/or chemo with LG‐PCa	−4.24725	Normal (−4.24725, 0.5774)	BC data
Log of PCa‐specific death rate‐WW/AS (untreated) with LG‐PCa	−5.79365	Normal (−5.79365, 0.1796)	BC data
Log of PCa‐specific death rate‐undetected LG‐PCa	−5.79365	Normal (−5.79365, 0.1796)	BC data and assumption
Log of PCa‐specific death rate‐any treatment with LG‐PCa	−5.83597	Normal (−5.83597, 0.2357)	BC data
Hazard ratio for death (treated vs. undetected) with LG‐PCa	0.5400	Lognormal (−0.75, 0.52)	^29^

Abbreviations: ADT, androgen deprivation therapy; AS, active surveillance; HG, high‐grade PCa; LG, low‐grade PCa; PCa, prostate cancer; WW, watchful waiting.

**TABLE 2:** Model cost parameters, point estimates, probability distribution, and data sources used in the cost‐effectiveness model.

Parameter	Point estimate (CAD)	Probability distribution^a^	References
Risk assessment tool	$270	Fixed	^19^
Biopsy	$1062	Gamma (8425, 8)	BC data
Radical prostatectomy, first year with PCa‐HG	$17,701	Normal (17,701, 214)	BC data
Radiotherapy, first year with PCa‐HG	$15,161	Normal (15,161, 232)	BC data
ADT and/or chemo, first year with PCa‐HG	$9413	Normal (9413, 1034)	BC data
WW/AS (untreated), first year with PCa‐HG	$3605	Normal (3605, 623)	BC data
Undetected PCa‐HG, first year	$3605	Normal (3605, 623)	BC data, assumption
Radical prostatectomy, first year with PCa‐LG	$16,106	Normal (16,106, 493)	BC data
Radiotherapy, first year with PCa‐LG	$10,219	Normal (10,219, 887)	BC data
ADT and/or chemo, first year with PCa‐LG	$9691	Normal (9691, 3687)	BC data
AS (untreated), first year with PCa‐LG	$1549	Normal (1549, 343)	BC data
Undetected PCa‐LG, first year	$1549	Normal (1549, 343)	BC data, assumption
Untreated in early stage (all grade)‐first year	$4232	Normal (4232, 325)	BC data
Untreated in advanced‐stage (all grade)‐first year	$8029	Normal (8029, 2403)	BC data
Radical prostatectomy, second year with PCa‐HG	$2220	Normal (2220, 216)	BC data
Radiotherapy, second year with PCa‐HG	$1589	Normal (1589, 233)	BC data
ADT and/or chemo, second year with PCa‐HG	$9462	Normal (9462, 888)	BC data
WW/AS (untreated), second year with PCa‐HG	$3918	Normal (3918, 502)	BC data
Undetected PCa‐HG, second year	$3918	Normal (3918, 502)	BC data, assumption
Radical prostatectomy, second year with PCa‐LG	$975	Normal (975, 320)	BC data
Radiotherapy, second year with PCa‐LG	$−177	Normal (−177, 883)	BC data
ADT and/or chemo, second year with PCa‐LG	$6496	Normal (6496, 2063)	BC data
AS (untreated), second year with PCa‐LG	$3189	Normal (3189, 308)	BC data
Undetected PCa‐LG, second year	$3189	Normal (3189, 308)	BC data, assumption
Untreated in early stage (all grade)‐second year	$2556	Normal (2556, 224)	BC data
Untreated in advanced‐stage (all grade)‐second year	$7598	Normal (7598, 2074)	BC data
Radical prostatectomy, annually year 3 and beyond with PCa‐HG	$1131	Normal (1131, 231)	BC data
Radiotherapy, annually year 3 and beyond with PCa‐HG	$1620	Normal (1620, 654)	BC data
ADT and/or chemo, annually year 3 and beyond with PCa‐HG	$6958	Normal (6958, 1017)	BC data
WW/AS (untreated), annually year 3 and beyond with PCa‐HG	$1355	Normal (1355, 463)	BC data
Undetected PCa‐HG, annually year 3 and beyond	$1355	Normal (1355, 463)	BC data, assumption
Radical prostatectomy, annually year 3 and beyond with PCa‐LG	$89	Normal (89, 317)	BC data
Radiotherapy, annually year 3 and beyond with PCa‐LG	$892	Normal (892, 789)	BC data
ADT and/or chemo, annually year 3 and beyond with PCa‐LG	$5327	Normal (5327, 2538)	BC data
WW/AS (untreated), annually year 3 and beyond with PCa‐LG	$1341	Normal (1341, 263)	BC data
Undetected PCa‐LG, annually year 3 and beyond	$1341	Normal (1341, 263)	BC data, assumption
Untreated in early stage (all grade, all PSA), annually year 3 and beyond	$1638	Normal (1638, 198)	BC data
Untreated in advanced‐stage (all grade, all PSA), annually year 3 and beyond	$2221	Normal (2221, 2492)	BC data
One year before death‐all groups	$9960	Normal (9960, 623)	BC data

Abbreviations: ADT, androgen deprivation therapy; AS, active surveillance; HG, high‐grade PCa; LG, low‐grade PCa; PCa, prostate cancer; WW, watchful waiting.

^**a**^ Numbers in parentheses represent the mean and standard deviation for the normal distribution, and the shape and scale for the gamma distribution.

#### 2.2.1 Data sources

Using linked administrative health data from BC Cancer and Population Data BC, we constructed a cohort of men aged ≥50 years with a diagnosis of PCa between 2010 and 2017 (Cohort 1 “cases”) and followed them from the date of diagnosis until death, the last date of observation, or December 31, 2019, whichever came first. We also created a cohort of PCa patients who were diagnosed with PCa after 1997 and died from PCa after 2010 (Cohort 2 “cases”) to facilitate the estimation of healthcare costs for the year before death. Controls who were No‐PCa individuals from the BC general population were matched to the cases in each cohort; details on matching are described in the Method S1 and our previous study.^37^ The study was approved by the behavioral research ethics board of the University of British Columbia (H20‐01258).

#### 2.2.2 PCa treatment distribution and transition probability

We estimated the probabilities of different follow‐up pathways based on Cohort 1 patients who had a PSA level in the gray zone (by grade at diagnosis and by treatment option within the first year of diagnosis). Using these data, we also calculated PCa‐specific mortality rates stratified by the grade of PCa in different health states to inform the transition probabilities. In the base case, men with undetected high‐grade and low‐grade PCa were assumed to have the same PCa‐specific mortality as untreated patients (WW/AS) in Cohort 1.

In scenario analyses, we tested the impact of this assumption and calculated the yearly probability of death of undetected high‐grade cases; we applied the hazard ratio of PCa‐specific mortality in treated patients versus patients under WW from the Bill‐Axelson et al.^29^ study to the estimated death rate of high‐grade PCa on any treatment from Cohort 1 (Table 1). In this scenario, the PCa‐specific mortality for the undetected low‐grade PCa was assumed to be the same as that for low‐grade patients receiving any treatment in Cohort 1. We also conducted another scenario in which the PCa‐specific mortality rate for both undetected low‐grade and high‐grade cases was calculated by applying the corresponding hazard ratio from Bill‐Axelson et al.^29^ to the death rate among patients on any treatment. Time‐dependent non‐PCa‐specific mortality rates were derived from Cohort 1 for high‐grade and low‐grade PCa patients and from the control group for the No‐PCa men, respectively.

#### 2.2.3 Detection power of the risk assessment tool

Many biomarkers (e.g., 4 K score, prostate health index, Progensa PCA3^38^, ^39^, ^40^) can be used as a RAT in this model. In the base case, we used the characteristics of SelectMDx, a urine‐based molecular biomarker,^41^ for the RAT strategy as an illustrative example to demonstrate the functionality of our model and to make our findings comparable to previous economic evaluation studies.^19^, ^20^, ^28^ A different combination of detection power was used as one scenario analysis.^42^ We also evaluated the impact of test characteristics on the model outcomes by varying the sensitivity and specificity parameters separately. The ranges were determined based on the detection power of PSA and the other existing RATs.^19^, ^20^, ^28^, ^43^, ^44^, ^45^

#### 2.2.4 Costs

Table 2 shows the estimated total healthcare costs attributable to PCa used in our model. For each PCa grade and treatment pathway, incremental costs attributable to PCa (difference in the total healthcare costs between the cases and matched controls) were estimated for the first year after the diagnosis, the second year, every year from the third year until 1 year before death (based on Cohort 1 and their controls), and 1 year before death (Cohort 2 and their controls).^37^ The incremental costs in these four time intervals were considered as costs attributable to PCa and included the incremental costs related to diagnostic tests and treatments incurred by the patients in each specific health state in the first year and beyond the first year.^37^ Figure S2 presents the mean annual incremental cost over time and supports the choice of modeling of the incremental cost by the time intervals. All costs were in 2021 Canadian dollars.

For the costs of undetected high‐grade and low‐grade PCa, we used the estimated costs of the corresponding untreated group in the base case. Based on Cohort 1, the ratio of advanced‐stage patients to early‐stage patients was much lower in untreated high‐grade patients than in all detected high‐grade patients. Thus, we tested a more conservative assumption in a scenario that assumed the undetected PCa cases had the same ratio of the advanced and early stages as all patients (treated and untreated) in Cohort 1. The cost of undetected cases was calculated based on this ratio and the respective costs of untreated patients in the advanced and early stages (Table 2).

We only considered the incremental cost of the intervention, that is, the cost of the RAT itself, because the tool is an add‐on to the current SOC. The cost of SOC itself cancels out when comparing strategies. The cost of biopsy was estimated using the Discharge Abstract Database from Population Data BC,^34^ which includes information on day procedures and hospitalizations.

#### 2.2.5 Utility weights

The disutility assigned to different diagnosis and treatment health states and their duration were primarily obtained from Heijnsdijk et al.^46^ (Table S1). In previous studies, the disutility assigned to WW/AS was assumed to be the same regardless of disease stage.^19^, ^20^, ^23^, ^28^ To improve this assumption, in the base case, we calculated the disutility assigned to the untreated groups (WW/AS, undetected high‐grade and low‐grade PCa) as a weighted average of disutility in advanced and early stages (Table S1; Method S1).^46^ We calculated the mean disutility for WW/AS based on the ratio of advanced and early stages among high‐ and low‐grade untreated patients (Table 1), respectively. In calculating the mean disutility for undetected PCa cases, the ratio of advanced and early stages in all diagnosed patients (separately for high‐grade and low‐grade) was used as the weight. We also evaluated the scenario in which the disutility of all untreated and undetected patients was assumed to be the disutility of WW/AS state for regardless of the disease stage.

### 2.3 Statistical analysis

Both costs and quality‐adjusted life years (QALYs) were discounted at 1.5% per year as recommended by the guidelines of the Canadian Agency for Drugs and Technologies in Health.^49^ Expected QALYs and costs in the base case and all scenarios were estimated through probabilistic analysis to address uncertainty around the model parameters. Probability distributions were assigned to the model parameters, and a Monte Carlo process modeling 10,000 simulations was conducted using randomly sampled values drawn from these distributions. Modeling and statistical analyses were performed with R (R Core Team, 2022).

#### 2.3.1 Scenario Analysis

We conducted a series of scenario analyses to assess how changes in input parameters and model assumptions could affect the outcomes. First, we evaluated the impact of PCa prevalence, cost of RAT, and RAT accuracy. While MRI is not yet the SOC for PCa in BC,^1^ we conducted a scenario using MRI as a RAT. In addition, since the ratio of high‐grade cases to all PCa patients in our sample was higher than most other studies from different settings, we examined how this ratio affected the outcomes. Lastly, we conducted some scenarios to examine the effect of our assumptions for undetected cases: (1) a weighted average of the cost of untreated patients in the advanced and early stages was used to calculate the cost of undetected cases; (2) we assumed the disutility of the WW/AS health state for all untreated and undetected patients, independent of the disease stage; (3) we used hazard ratio from the Bill‐Axelson et al.^29^ study to estimate survival rates for undiagnosed low‐grade and high‐grade PCa.

## 3 RESULTS

### 3.1 Base case analysis

**FIGURE 1:**
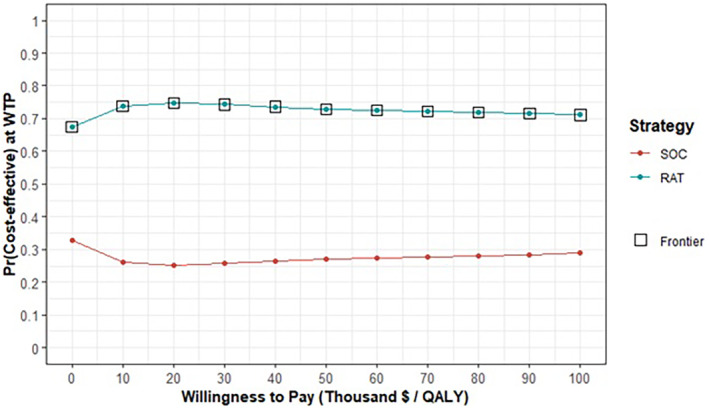
Cost‐effectiveness acceptability curve for the base case. QALY, quality‐adjusted life years; RAT, risk assessment tool; SOC, standard of care; WTP, willingness‐to‐pay.

Results of the base case analysis are presented in Table 3, Figure 1, and Figure S3. As shown, the RAT, based on the characteristics of SelectMDx, was a dominant strategy associated with an average cost saving of $44 and a QALY gain of 0.00253 over 18 years of follow‐up. The cost‐effectiveness acceptability curve in Figure 1 shows there is a 73% probability that using a RAT to guide biopsy is cost‐effective compared to the SOC at the willingness‐to‐pay threshold of $50,000/QALY gain.

**TABLE 3:** Mean and incremental costs, mean and incremental quality‐adjusted life‐years and incremental cost‐effectiveness ratios of the base case and scenario analyses.

Scenario	SOC		RAT		Incremental			Probability of RAT being cost‐effective	
	Costs $	QALY	Costs $	QALY	Costs $	QALY	ICER (RAT compared to SOC) $/QALY	@ WTP $50,000/QALY	@ WTP $100,000/QALY
Base case	8462	13.6593	8418	13.6618	−44	0.00253	Dominant	73%	71%
*SA: PCa prevalence (base case PCa prevalence = 0.25)*									
0.134^57^	5028	13.3853	4831	13.3884	−197	0.00305	Dominant	99%	96%
0.21^48^	7278	13.5648	7181	13.5675	−97	0.00271	Dominant	84%	80%
0.28^58^	9350	13.7301	9346	13.7325	−4	0.00240	Dominant	65%	66%
0.32^56^	10,534	13.8246	10,583	13.8268	49	0.00222	22,204	57%	59%
*SA: cost of RAT (base case cost of RAT = 270)*									
$670	8462	13.6593	8808	13.6618	346	0.00253	136,791	21%	42%
$570	8462	13.6593	8711	13.6618	249	0.00253	98,271	32%	50%
$470	8462	13.6593	8613	13.6618	151	0.00253	59,571	46%	57%
$370	8462	13.6593	8516	13.6618	54	0.00253	21,230	60%	64%
$314	8462	13.6593	8461	13.6618	−1	0.00253	Dominant	68%	68%
$170	8462	13.6593	8321	13.6618	−141	0.00253	Dominant	83.5%	78%
*SA: RAT specificity for No‐PCa (base case specificity for No‐PCa = 0.608)*									
0.758	8462	13.6593	8299	13.6625	−163	0.00320	Dominant	88%	82%
0.708	8462	13.6593	8338	13.6623	−124	0.00298	Dominant	84%	79%
0.658	8462	13.6593	8378	13.6620	−84	0.00275	Dominant	79%	75%
0.558	8462	13.6593	8458	13.6616	−4	0.00230	Dominant	66%	67%
0.508	8462	13.6593	8498	13.6614	36	0.00208	17,266	59%	62%
0.458	8462	13.6593	8538	13.6611	76	0.00185	40,835	52%	58%
*SA: RAT specificity for LG‐PCa (base case specificity for LG‐PCa = 0.336)*									
0.436	8462	13.6593	8500	13.6709	36	0.01163	3311	95%	97%
0.386	8462	13.6593	8459	13.6664	−3	0.00708	Dominant	88%	90%
0.286	8462	13.6593	8377	13.6573	−85	−0.00202	^a^	47%	40%
*SA: RAT sensitivity for HG‐PCa (base case sensitivity for HG‐PCa = 0.957)*									
0.97	8462	13.6593	8447	13.6711	−15	0.01184	Dominant	99.5%	99.6%
0.96	8462	13.6593	8425	13.6640	−37	0.00468	Dominant	84%	84%
0.95	8462	13.6593	8403	13.6568	−59	−0.00248	^a^	40%	36%
0.94	8462	13.6593	8380	13.6496	−82	−0.00964	^a^	11%	1%
*SA: RAT accuracy*									
RAT sensitivity for HG = 0.90, specificity for LG = 0.35, specificity for No‐PCa = 0.58^42^	8462	13.6593	8325	13.6221	−137	−0.03714	^a^	0.05%	0.02%
*SA: MRI as a RAT*									
Cost = 1003^25^ ^,^ ^b^ sensitivity for HG = 0.901, specificity for LG = 0.251, specificity for No‐PCa = 0.734^22^	8462	13.6593	8835	13.6145	373	−0.04480	Dominated	0%	0%
*SA: Ratio of high‐grade PCa: Probability of high‐grade = 0.5* 19, 20, 28									
RAT sensitivity for HG same as base case =0.957	6958	13.4294	7166	13.4668	208	0.03741	5552	100%	100%
0.94	6958	13.4294	7140	13.4585	182	0.02903	6263	99.9%	99.99%
0.90	6958	13.4294	7079	13.4387	121	0.00931	12,985	77%	82%
0.885	6958	13.4294	7056	13.4313	98	0.00191	51,203	49%	53%
0.88	6958	13.4294	7049	13.4289	91	−0.00055	Dominated	40%	43%
0.87	6958	13.4294	7033	13.4240	75	−0.00548	Dominated	25%	26%
0.86	6958	13.4294	7018	13.4190	60	−0.01041	Dominated	14%	14%
0.85	6958	13.4294	7003	13.4141	45	−0.01534	Dominated	0. 8%	0.7%
*SA: Assumptions about undetected cases*									
Cost of undetected HG and LG as weighted average of advanced and early stage	8462	13.6593	8609	13. 6618	147	0.00253	58,294	47%	58%
Disutility of WW/AS^19^, ^20^, ^46^ was for all untreated patients (WW/AS, undetected HG and LG) during the entire follow‐up	8462	13.6601	8418	13.6644	−44	0.00428	Dominant	83%	82%
Mortality rate of undetected LG same as all treated LG, mortality rate of undetected HG calculated using a hazard ratio^29^	8462	13.6593	8417	13.6602	−45	0.00090	Dominant	61%	59%
Mortality rate of both undetected LG and HG cases calculated using a hazard ratio^29^	8462	13.6593	8410	13.6499	−52	−0.00942	^a^	24%	22%

Abbreviations: AS, active surveillance; HG, high‐grade PCa; LG, low‐grade PCa; MRI, magnetic resonance imaging; PCa, prostate cancer; QALY, quality‐adjusted life‐year; RAT, risk assessment tool; SA, scenario analysis; SOC, standard of care; WTP, willingness‐to‐pay; WW, watchful waiting.

^a^ RAT has a lower cost but less QALYs compared to standard of care.

^b^ Converted to 2021 CAD using the Consumer Price Index for health and personal care reported by Statistics Canada.^59^

### 3.2 Scenario analyses

**FIGURE 2:**
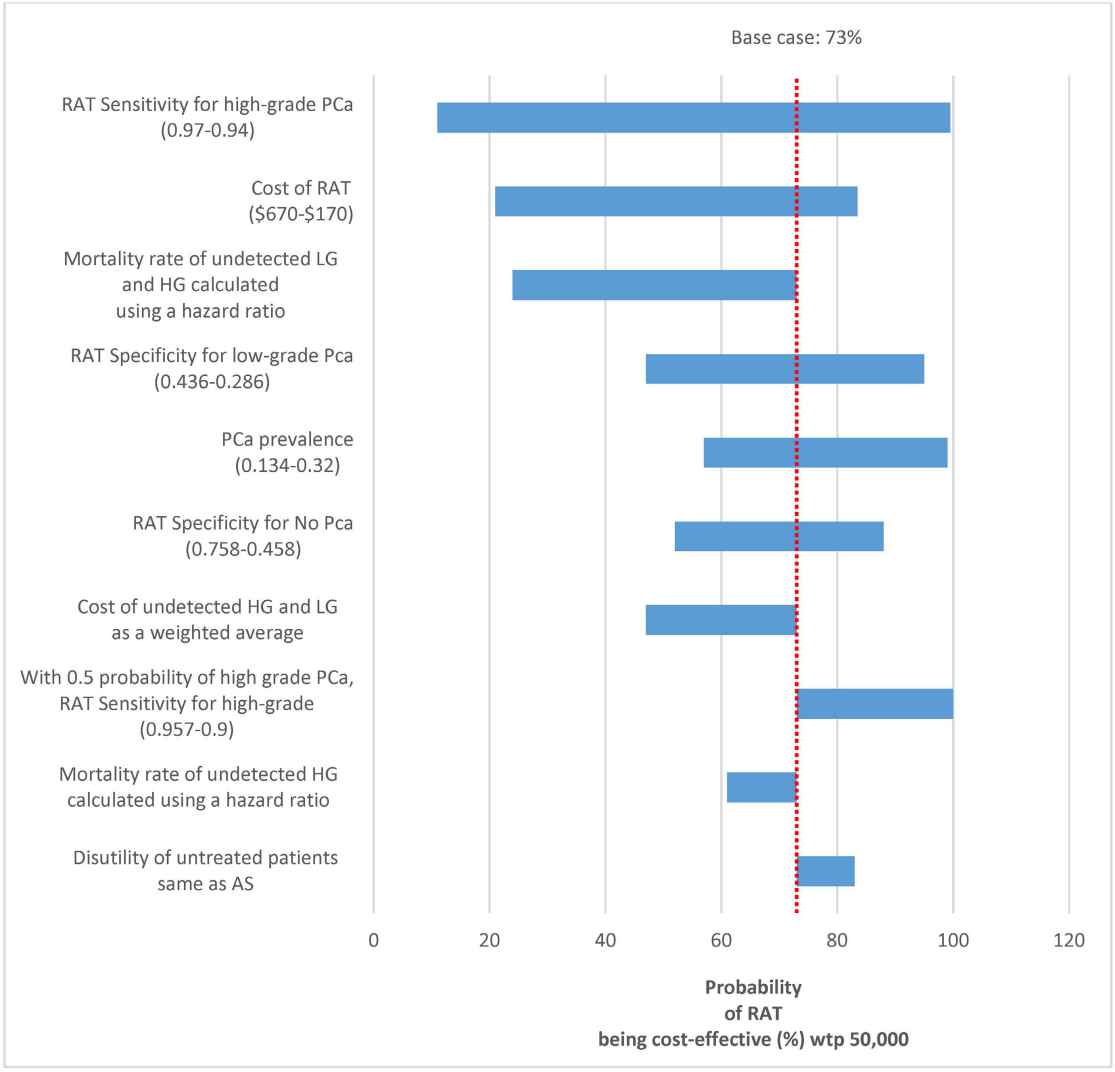
Results of scenario analysis. AS, active surveillance; HG, high‐grade PCa; LG, low‐grade PCa; PCa, prostate cancer; PCa, prostate cancer; QALY, quality‐adjusted life years; RAT, risk assessment tool; SA, scenario analysis; SOC, standard of care; WTP, willingness‐to‐pay; WW, watchful waiting.

Table 3, Figure 2, and Results S1 present the findings of scenario analyses evaluating the effects of changing the main model parameters and assumptions. Overall, scenario analyses showed that model outcomes were most sensitive to the RAT detection rate of high‐grade cancer. The cost‐effectiveness outcome could also be affected by the RAT cost, the prevalence of PCa, the percentage of high‐grade PCa, and different assumptions about the cost and survival of the undetected cases.

## 4 DISCUSSION

This study provides a general modeling framework to evaluate the impact on outcome and resource use of using any new RAT to inform PCa diagnosis decisions. Our cost‐effectiveness analysis demonstrated that incorporating an accurate tool in the initial diagnosis of PCa in men with PSA levels in the gray zone can be a cost‐effective (or dominant) strategy by avoiding unnecessary biopsies and overtreatment. Our extensive set of scenario analyses further revealed that the cost‐effectiveness results were sensitive to the tool's accuracy, especially the detection rate for high‐grade PCa. The results also showed that, for a RAT to be cost‐effective, its sensitivity must be relatively high (>0.955); however, this sensitivity could change with the different proportions of high‐grade PCa in the sample. The cost of RAT and PCa prevalence can also impact whether RAT is cost‐effective compared to PSA alone. The results showed that RAT can be a dominant strategy at costs below $314, given other RAT characteristics and assumptions made in the base case. We also found that the cost‐effectiveness results were impacted by the different assumptions on cost and survival rate of the undetected cases resulting from using a RAT to guide biopsy.

The results of scenario analyses for evaluating the impact of changes in model inputs align with previous studies conducted to assess the value of specific biomarkers or other diagnostic methods.^19^, ^20^, ^21^, ^26^, ^28^, ^47^, ^48^ For example, Dijkstra et al.^19^ and Govers et al.^20^, ^28^ found that using SelectMDx was a dominant strategy, and the results were sensitive to the test's detecting power. Similar results were found by three other economic evaluations of the use of MRI to guide biopsy.^24^, ^50^, ^51^ In other research, the cost of a new RAT was also mentioned as a parameter that could affect the cost‐effectiveness result.^21^, ^47^, ^52^ However, previous analyses have significant heterogeneity in disease pathways and modeling approaches.^26^ As such, in the current study, we aimed to provide a general model or framework to evaluate any RAT (even tools under development) and facilitate direct comparisons between different diagnostic methods. In this framework, we developed and conducted a decision analytical model. Through collaboration with patient partners and clinicians and also using administrative data to inform model parameters, we attempted to consider uncertainties and assess the effects of RATs on QALYs and costs in a variety of scenarios that might represent different existing or novel RATs.

This study has some limitations. First, the cost‐effectiveness analysis was performed from the BC healthcare payer's perspective, and BC data were used to inform the model, which might limit the generalizability of results to other provinces or countries. Nevertheless, our proposed modeling framework can be easily modified to analyze the cost and effectiveness of using a new RAT to direct PCa diagnosis in another setting. Second, the SOC in the model illustrates a strategy of using PSA alone to guide biopsy and thus, may not fully represent current practice for PCa diagnosis (e.g., addition of MRI, sequential PSA testing for some individuals). However, since we estimated our cost and mortality model parameters using the population‐based administrative data, the costs of MRI or sequential PSA testing, if any, and their impact have been implicitly considered. Furthermore, any strategy different from the SOC could be represented as a RAT in our model and scenario analyses to evaluate its cost‐effectiveness. Third, although we attempted to minimize the confounding effect using the matching method when estimating incremental costs and mortality attributable to PC, these estimates may still be confounded. For example, we matched on the number of comorbidities rather than the exact conditions. Lastly, in our study, we employed a time‐dependent cohort state‐transition model. While the existing microsimulations model, such as the Fred Hutchinson Cancer Research Center (FHCRC) model,^53^, ^54^, ^55^ has its merits and could potentially provide a valuable perspective, its implementation requires extensive data which were not readily available for our study population.

Importantly, a key strength of this study was taking a more conservative approach by considering the impact of undetected PCa on patient survival, quality of life and costs. The decrease in utility or associated costs of PCa cases missing due to false negative RAT results was not taken into account in some previous studies.^19^, ^20^, ^28^ However, in this study, we considered the disutility of undetected cases due to the delay in treatment and separately calculated this disutility for men with advanced and early stages of PCa. We also accounted for the potential costs associated with undetected high‐grade and low‐grade PCa. Moreover, by assuming different PCa‐specific mortality rates, we explored the impact of missing cases on patient survival. Our scenario analyses showed that the cost‐effectiveness results were sensitive to some of these assumptions, which highlights the importance of such considerations.

Another key strength of this work is our collaboration with patient partners and clinicians to define the diagnostic pathway as the basis for our economic model. Both stakeholder groups emphasized the need for a better RAT than PSA alone to guide biopsy decisions, especially for the PSA gray zone, and provided unique insights on the diversity and complexity of diagnosis and treatment pathways. Another important strength is our use of real‐world, whole‐population administrative health data from multiple data sources to inform the model. This provided a more accurate reflection of the current practice and diversity of treatment pathways in the cost‐effectiveness analysis. Lastly, we characterized uncertainty in the model input parameters as a distribution and conducted a probabilistic analysis for the base case and all 36 scenarios through Monte Carlo simulation.

## 5 CONCLUSION

This study found that a new or emerging more accurate RAT to guide clinical decisions about biopsy in men at risk of PCa can be a dominant strategy compared to the SOC. However, the cost‐effectiveness decisions about a RAT are sensitive to its cost and accuracy, especially the detection rate for high‐grade cancers. This study also provides a general economic evaluation framework to analyze the cost‐effectiveness of any novel marker to improve the diagnosis process of PCa.

## AUTHOR CONTRIBUTIONS

**Tima Mohammadi:** Conceptualization (equal); data curation (supporting); formal analysis (lead); investigation (lead); methodology (lead); software (lead); validation (lead); visualization (lead); writing – original draft (lead); writing – review and editing (lead). **Daphne P. Guh:** Conceptualization (equal); data curation (supporting); formal analysis (lead); investigation (lead); methodology (lead); software (lead); validation (equal); writing – review and editing (equal). **Alexander CT Tam:** Data curation (supporting); investigation (supporting); project administration (equal); writing – review and editing (equal). **Reka E. Pataky:** Data curation (supporting); investigation (equal); methodology (equal); validation (equal); writing – review and editing (equal). **Peter Black:** Investigation (equal); resources (supporting); validation (supporting); writing – review and editing (equal). **Alan So:** Investigation (equal); resources (supporting); validation (supporting); writing – review and editing (equal). **Larry Lynd:** Conceptualization (lead); funding acquisition (lead); methodology (equal); project administration (supporting); resources (equal); writing – review and editing (equal). **Wei Zhang:** Conceptualization (lead); data curation (lead); funding acquisition (lead); investigation (equal); methodology (equal); project administration (equal); resources (equal); supervision (equal); validation (equal); writing – original draft (supporting); writing – review and editing (equal). **Annalijn I. Conklin:** Conceptualization (lead); data curation (lead); funding acquisition (lead); investigation (equal); methodology (equal); project administration (equal); resources (equal); supervision (equal); writing – review and editing (equal).

## FUNDING INFORMATION

This work was supported by a GlycoNet Collaborative Team Grant from the Canadian Glycomics Network, project titled ‘The early study: ECONOMIC evaluation of a novel prostate cancer glycan‐based diagnostic tool (CR‐01)’, with matched funding from the Michael Smith Health Research BC, the University of British Columbia Faculty of Pharmaceutical Sciences, and the Centre for Health Evaluation and Outcome Sciences. WZ and AIC are independently supported by the Michael Smith Health Research BC Scholar award. RP is independently supported by the Canadian centre for Applied Research in Cancer Control (ARCC), which is funded by the Canadian Cancer Society. ACTT was previously independently supported by the Canadian Institutes of Health Research Canada Graduate Scholarship‐Master's at the time this research was conducted. The funder did not play a role in the design of the study; the collection, analysis, and interpretation of the data; the writing of the manuscript; and the decision to submit the manuscript for publication.

## CONFLICT OF INTEREST STATEMENT

The authors declare no conflict of interest.

## ETHICS STATEMENT

The study was approved by the behavioral research ethics board of the University of British Columbia (H20‐01258).

## Supporting information


Appendix S1.

## Data Availability

The data that supports the findings of this study are available from Population Data BC (https://www.popdata.bc.ca/), but restrictions apply to the availability of these data. The data were used under approval and research agreements with the Data Stewards, and so are not publicly available. Access to data provided by the Data Steward(s) is subject to approval, but can be requested for research projects through the Data Steward(s) or their designated service providers. All inferences, opinions, and conclusions drawn in this publication are those of the author(s), and do not reflect the opinions or policies of the Data Steward(s).
