# Supplementary material for: Economic evaluation of prostate cancer risk assessment methods: A cost‐effectiveness analysis using population data
Source: Cancer Med. 2023 Sep 23;12(19):20106–18. doi: 10.1002/cam4.6587 (PMC10587968; doi:10.1002/cam4.6587)
Supplement: Supplementary file 1 — Appendix S1. [file CAM4-12-20106-s001.docx]

**Online-only Supplemental Material**

[eMethod 2](#_Toc131679359)

[eResults 3](#_Toc131679360)

[eFigure1. Patient diagnosis and treatment pathway for PCa as the structure of the cost-effectiveness model 5](#_Toc131679361)

eFigure2. Mean cost by year by primary treatment among PSA 3 to 10

[eFigure 3. Cost-effectiveness plane for the base case 6](#_Toc131679362)

[eTable 1. Disutility Weights, Point Estimate, Probability Distributions, and Data Sources Used in the Cost-Effectiveness Model 7](#_Toc131679363)

eFile: Model R code

[References 8](#_Toc131679364)

# eMethod

**Disutility weights**

For the advanced stage, we calculated the yearly disutility based on the average annual estimate of the disutility of WW/AS state for 186 months and the disutility of palliative therapy for 30 months (eTable1). The disutility of the early stage was assumed to be the same as the disutility of WW/AS state for the entire 18-year follow-up period.

**Matching**

For the purpose of estimating costs, up to 2 controls for the cases in Cohort 1 and Cohort 2 were matched to No-PCa individuals from the BC general population by age (±2 years), health service delivery area (16 areas covering the province of BC used to provide program delivery and services), neighbourhood income quintile (Quintile of Annual Income Per Person Equivalent), Elixhauser Comorbidity Index ^64^ (0, 1, 2, 3+) and the year of last follow-up. For the purpose of estimating mortality, up to 5 controls for the cases in Cohort 1were matched to No-PCa individuals from the BC general population by age (±2 years), health service delivery area, neighbourhood income quintile and Elixhauser Comorbidity Index.

**Patient partner engagement**

To recruit patient partners, we created a posting on an online directory of local research opportunities, REACH BC. We formed a patient advisory group consisting of 5 men who had lived experience with PCa screening and diagnostic tests. The goals of engaging with the patient advisory group were to: (1) share the clinical pathway diagram that we had constructed based on local clinical guidelines, literature, and clinical expertise, and (2) identify where the pathway differed on an individual patient level. Patient partners were compensated for their time.

# eResults

**Scenario Analysis Results**

First, we evaluated the impact of changes in the prevalence of PCa in men with PSA in the grey zone over a plausible range. The range was determined based on the estimated PCa prevalence in different studies.^60–62,65^ As shown in Table 3, the probability of RAT being cost-effective compared to SOC decreases with increasing PCa prevalence.

Second, we ran the model for RAT costs ranging from $170 to $670. As expected, with decreasing tool costs, using RAT to guide biopsy became more cost-effective. Based on the point estimates of cost and QALY differences, the RAT strategy was dominant when the tool cost less than $314. The probability of the RAT strategy being cost-effective increased from 68% at $314 to 83.5% at $170 at the willingness-to-pay (WTP) threshold of $50,000/QALY.

Third, we evaluated the impact of changing the RAT accuracy characteristics: the model outcomes were most sensitive to the detection rate of high-grade cancer. As the sensitivity for high-grade changed from 0.97 to 0.94, the probability of RAT being cost-effective at WTP $50,000/QALY dropped from 99% to 11%. Keeping the same sensitivity, shifting the specificity for low-grade PCa from 0.436 to 0.286, and the specificity for No-PCa from 0.758 to 0.458, also decreased the probability of RAT being cost-effective strategy but the decrease was not substantial.

Fourth, since the ratio of high grade to all PCa patients in our cohort was higher compared to most other studies from different settings, we examined the impact of this ratio on the outcomes. We ran the scenario with RAT characteristics similar to the base case and then changed the RAT sensitivity for high-grade PCa because it was found to have a substantial impact on the results in the scenario analyses above. As shown in Table 3, given the ratio of high-grade to total PCa equal to 50%, the results were much less sensitive to the detection power of RAT, with RAT being cost-effective at the RAT detection power for high-grade PCa ≥ 0.885.

Fifth, we tested the effect of our assumption on the cost of undetected (undetected) PCa patients. In the base case, we assumed that the cost of the first year, second year and third year until one year before death for this group, was similar to the average of untreated patients (WW/AS). We further considered a scenario in which the cost of undetected high-grade cases was assumed to be a weighted average of the cost of untreated patients in advanced and early stages (using the ratio of advanced to early stage in all detected high-grade PCa). Taking this conservative assumption increased the cost of the RAT strategy, bringing the ICER to $58,294/QALY gained. This strategy had a 47% probability of being cost-effective compared to SOC (at WTP $50,000/ QALY).

Sixth, the results of scenario analysis showed that considering the disutility of the WW/AS state for all untreated patients regardless of the disease stage did not significantly impact the outcome of the models.

Finally, we assumed that undetected low-grade PCa would not impact survival. Cancer-specific mortality rates in undetected low-grade PCa were therefore considered to be similar to the treated low-grade patients. But a hazard ratio was applied to the mortality rate of the treated high-grade patients to calculate the PCa-specific mortality rate for the undetected high-grade PCa. Using this assumption, the probability of a RAT strategy being cost-effective compared to SOC decreased to 61%. When the hazard ratio was applied to the PCa-specific mortality rate among both undetected high- and low-grade PCa, using the RAT was only cost-effective in 24% of the iterations; based on the point estimate, using RAT had lower cost but also less QALYs (Table 3).


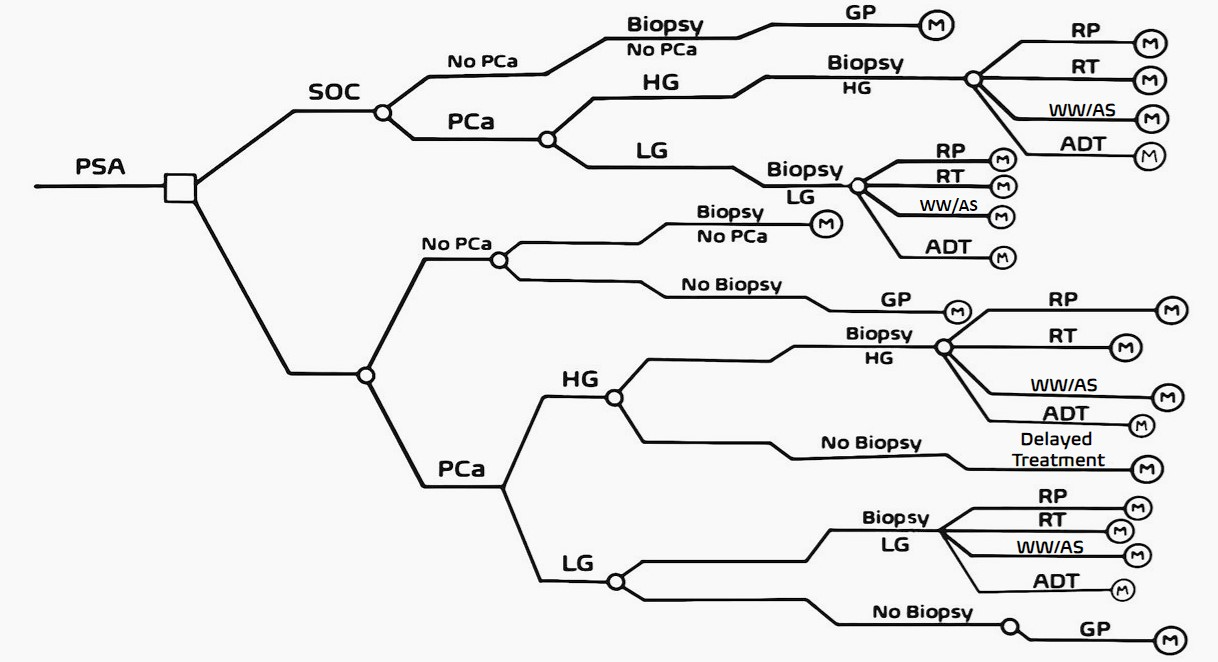


# eFigure1. Patient diagnosis and treatment pathway for PCa as the structure of the cost-effectiveness model

Abbreviations: PSA, prostate-specific antigen; PCa, prostate cancer; SOC, standard of care; RAT, risk assessment tool; HG, high-grade PCa; LG, low-grade PCa; AS, active surveillance; WW, watchful waiting; ADT, androgen deprivation therapy; RP, radical prostatectomy; RT, radiotherapy; GP, general practitioner.

# eFigure2. Mean cost by year by primary treatment among PSA 3 to 10


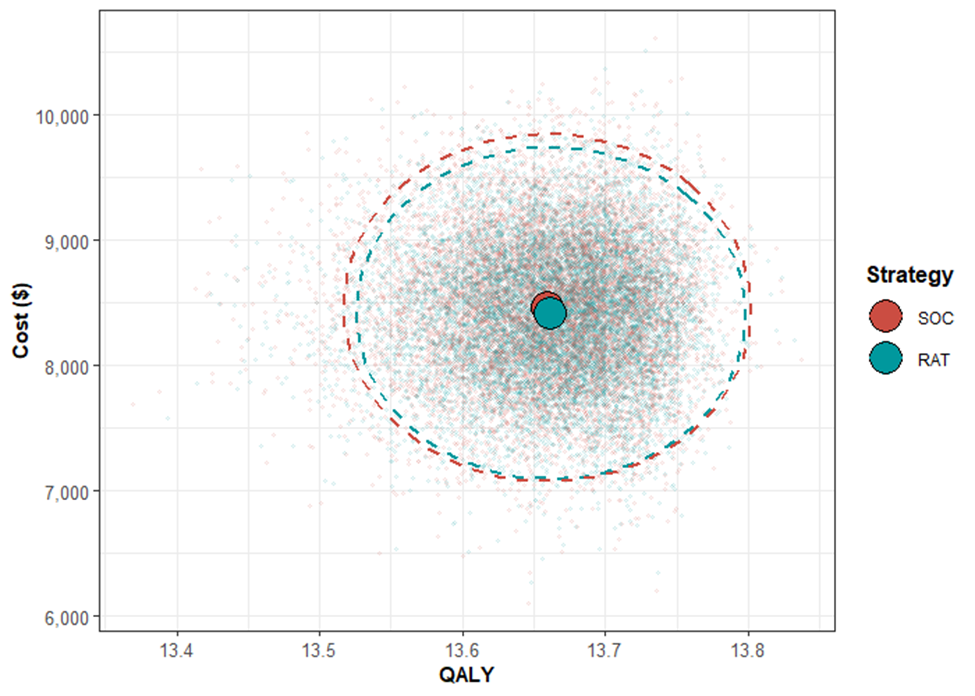


# eFigure3. Cost-effectiveness plane for the base case

Abbreviations: QALY, quality-adjusted life years; SOC, standard of care; RAT, risk assessment tool.

# eTable 1. Disutility Weights, Point Estimate, Probability Distributions, and Data Sources Used in the Cost-Effectiveness Model

| **Parameter** | **Value** | **Probability Distribution** | **References** |
| --- | --- | --- | --- |
| Biopsy | 0.006 (First year) | Beta (34.4,5697) | ^48^ |
| PCa diagnosis | 0.017 (First year) | Beta (53.9,3115.3) | ^48^ |
| Radical prostatectomy | 0.228 (First year) | Beta (7.9,26.6) | ^48^ |
| Radiotherapy | 0.247 (First year) | Beta (6.9,21.1) | ^48^ |
| ADT and/or chemo | 0.17 (First year and beyond) | Beta (25.4,124.2) | ^66^ |
| WW/AS | 0.03 (First year and beyond) | Beta (3.7,119.4) | ^48^ |
| Post recovery period (radical prostatectomy and radiotherapy) | 0.05 (After first year) | Beta (3.6, 68.4) | ^48^ |
| Palliative therapy | 0.4 (After first year) | Beta (2.4,3.7) | ^48^ |

Abbreviations: PCa, prostate cancer; AS, active surveillance; WW, watchful waiting; ADT, androgen deprivation therapy.

**eFile.Model R Code**

############

df_out_ce<-function(n.t){

p.HG<-rbeta(n_sim,p.HG1,p.HG2)

p.LG<-1-p.HG

p.HG.RP<-rbeta(n_sim,p.HG.RP1,p.HG.RP2)

p.HG.RT<-rbeta(n_sim,p.HG.RT1,p.HG.RT2)

p.HG.ADT<-rbeta(n_sim,p.HG.ADT1,p.HG.ADT2)

p.HG.WW<-1-(p.HG.RP+p.HG.RT+p.HG.ADT)

p.LG.RP<-rbeta(n_sim,p.LG.RP1,p.LG.RP2)

p.LG.RT<-rbeta(n_sim,p.LG.RT1,p.LG.RT2)

p.LG.ADT<-rbeta(n_sim,p.LG.ADT1,p.LG.ADT2)

p.LG.AS<-1-(p.LG.RP+p.LG.RT+p.LG.ADT)

logr.LN2D.HG.RP<-rnorm(n_sim,LN2D.HG.RP1,LN2D.HG.RP2)

r.LN2D.HG.RP<-exp(logr.LN2D.HG.RP)

P.LN2D.HG.RP<-1-exp(-r.LN2D.HG.RP)

logr.LN2D.HG.RT<-rnorm(n_sim,LN2D.HG.RT1,LN2D.HG.RT2)

r.LN2D.HG.RT<-exp(logr.LN2D.HG.RT)

P.LN2D.HG.RT<-1-exp(-r.LN2D.HG.RT)

logr.LN2D.HG.ADT<-rnorm(n_sim,LN2D.HG.ADT1,LN2D.HG.ADT2)

r.LN2D.HG.ADT<-exp(logr.LN2D.HG.ADT)

P.LN2D.HG.ADT<-1-exp(-r.LN2D.HG.ADT)

logr.LN2D.HG.WW<-rnorm(n_sim,LN2D.HG.WW1,LN2D.HG.WW2)

r.LN2D.HG.WW<-exp(logr.LN2D.HG.WW)

P.LN2D.HG.WW<-1-exp(-r.LN2D.HG.WW)

logr.LN2D.HG.allT<-rnorm(n_sim,LN2D.HG.allT1,LN2D.HG.allT2)

r.HG.allT<-exp(logr.LN2D.HG.allT)

hr.HG<-rlnorm(n_sim,hr.HG1,hr.HG2)

P.LN2D.HG.M<-P.LN2D.HG.WW

logr.LN2D.LG.RP<-rnorm(n_sim,LN2D.LG.RP1,LN2D.LG.RP2)

r.LN2D.LG.RP<-exp(logr.LN2D.LG.RP)

P.LN2D.LG.RP<-1-exp(-r.LN2D.LG.RP)

logr.LN2D.LG.RT<-rnorm(n_sim,LN2D.LG.RT1,LN2D.LG.RT2)

r.LN2D.LG.RT<-exp(logr.LN2D.LG.RT)

P.LN2D.LG.RT<-1-exp(-r.LN2D.LG.RT)

logr.LN2D.LG.ADT<-rnorm(n_sim,LN2D.LG.ADT1,LN2D.LG.ADT2)

r.LN2D.LG.ADT<-exp(logr.LN2D.LG.ADT)

P.LN2D.LG.ADT<-1-exp(-r.LN2D.LG.ADT)

logr.LN2D.LG.AS<-rnorm(n_sim,LN2D.LG.AS1,LN2D.LG.AS2)

r.LN2D.LG.AS<-exp(logr.LN2D.LG.AS)

P.LN2D.LG.AS<-1-exp(-r.LN2D.LG.AS)

logr.LN2D.LG.allT<-rnorm(n_sim,LN2D.LG.allT1,LN2D.LG.allT2)

r.LG.allT<-exp(logr.LN2D.LG.allT)

hr.LG<-rlnorm(n_sim,Lr.LG1,Lr.LG2)

P.LN2D.LG.M<-P.LN2D.LG.AS

#######

CRP.Y1.HG<-rnorm(n_sim,CRP.Y1.HG1,CRP.Y1.HG2)

CRT.Y1.HG<-rnorm(n_sim,CRT.Y1.HG1,CRT.Y1.HG2)

CADT.Y1.HG<-rnorm(n_sim,CADT.Y1.HG1,CADT.Y1.HG2)

CAS.Y1.HG<-rnorm(n_sim,CAS.Y1.HG1,CAS.Y1.HG2)

CMD.Y1.HG<-rnorm(n_sim,CAS.Y1.HG1,CAS.Y1.HG2)

CRP.Y1.LG<-rnorm(n_sim,CRP.Y1.LG1,CRP.Y1.LG2)

CRT.Y1.LG<-rnorm(n_sim,CRT.Y1.LG1,CRT.Y1.LG2)

CADT.Y1.LG<-rnorm(n_sim,CADT.Y1.LG1,CADT.Y1.LG2)

CAS.Y1.LG<-rnorm(n_sim,CAS.Y1.LG1,CAS.Y1.LG2)

CMD.Y1.LG<-rnorm(n_sim,CAS.Y1.LG1,CAS.Y1.LG2)

##################

CRP.Y2.HG<-rnorm(n_sim,CRP.Y2.HG1,CRP.Y2.HG2)

CRT.Y2.HG<-rnorm(n_sim,CRT.Y2.HG1,CRT.Y2.HG2)

CADT.Y2.HG<-rnorm(n_sim,CADT.Y2.HG1,CADT.Y2.HG2)

CAS.Y2.HG<-rnorm(n_sim,CAS.Y2.HG1,CAS.Y2.HG2)

CMD.Y2.HG<-rnorm(n_sim,CAS.Y2.HG1,CAS.Y2.HG2)

CRP.Y2.LG<-rnorm(n_sim,CRP.Y2.LG1,CRP.Y2.LG2)

CRT.Y2.LG<-rnorm(n_sim,CRT.Y2.LG1,CRT.Y2.LG2)

CADT.Y2.LG<-rnorm(n_sim,CADT.Y2.LG1,CADT.Y2.LG2)

CAS.Y2.LG<-rnorm(n_sim,CAS.Y2.LG1,CAS.Y2.LG2)

CMD.Y2.LG<-rnorm(n_sim,CAS.Y2.LG1,CAS.Y2.LG2)

############

CLN.HG.RP<-rnorm(n_sim,CLN.HG.RP1,CLN.HG.RP2)

CLN.HG.RT<-rnorm(n_sim,CLN.HG.RT1,CLN.HG.RT2)

CLN.HG.ADT<-rnorm(n_sim,CLN.HG.ADT1,CLN.HG.ADT2)

CLN.HG.AS<-rnorm(n_sim,CLN.HG.AS1,CLN.HG.AS2)

CLN.HG.MD<-rnorm(n_sim, CLN.HG.AS1, CLN.HG.AS2)

CLN.LG.RP<-rnorm(n_sim,CLN.LG.RP1,CLN.LG.RP2)

CLN.LG.RT<-rnorm(n_sim,CLN.LG.RT1,CLN.LG.RT2)

CLN.LG.ADT<-rnorm(n_sim,CLN.LG.ADT1,CLN.LG.ADT2)

CLN.LG.AS<-rnorm(n_sim,CLN.LG.AS1,CLN.LG.AS2)

CLN.LG.MD<-rnorm(n_sim,CLN.LG.AS1,CLN.LG.AS2)

############

Cdea.Y<-rnorm(n_sim,Cdea.Y1,Cdea.Y2)

CBiopsy<-rgamma(n_sim,CBiopsy1,CBiopsy2)

##########

dURP<-rbeta(n_sim,dURP1,dURP2)

dURT<-rbeta(n_sim,dURT1,dURT2)

dUAS<-rbeta(n_sim,dUAS1,dUAS2)

dUADT<-rbeta(n_sim,dUADT1,dUADT2)

dUBiopsy<-rbeta(n_sim,dUBiopsy1,dUBiopsy2)

dUPCa<-rbeta(n_sim,dUPCa1,dUPCa2)

dULN.RP<-rbeta(n_sim,dULN.RP1,dULN.RP2)

dULN.RT<-rbeta(n_sim,dULN.RT1,dULN.RT2)

dULN.AS<-rbeta(n_sim,dULN.AS1,dULN.AS2)

dULN.ADT<-rbeta(n_sim,dULN.ADT1,dULN.ADT2)

dULN.PaT<-rbeta(n_sim,dULN.PaT1,dULN.PaT2)

P.LN2D.NOPCa<-p_mor_age_NoPCa

t.LN.HG.RT <- numeric()

t.LN.HG.RT[2] <- P.LN2D.HG.RT

t.LN.HG.RT[1] <- 1 - t.LN.HG.RT[2]

t.LN.HG.RP <- numeric()

t.LN.HG.RP[2] <- P.LN2D.HG.RP

t.LN.HG.RP[1] <- 1 - t.LN.HG.RP[2]

t.LN.HG.ADT <- numeric()

t.LN.HG.ADT[2] <- P.LN2D.HG.ADT

t.LN.HG.ADT[1] <- 1 - t.LN.HG.ADT[2]

t.LN.HG.WW <- numeric()

t.LN.HG.WW[2] <- P.LN2D.HG.WW

t.LN.HG.WW[1] <- 1 - t.LN.HG.WW[2]

t.LN.HG.M <- numeric()

t.LN.HG.M[2] <- P.LN2D.HG.M

t.LN.HG.M[1] <- 1 - t.LN.HG.M[2]

t.LN.LG.RT <- numeric()

t.LN.LG.RT[2] <- P.LN2D.LG.RT

t.LN.LG.RT[1] <- 1 - t.LN.LG.RT[2]

t.LN.LG.RP <- numeric()

t.LN.LG.RP[2] <- P.LN2D.LG.RP

t.LN.LG.RP[1] <- 1 - t.LN.LG.RP[2]

t.LN.LG.ADT <- numeric()

t.LN.LG.ADT[2] <- P.LN2D.LG.ADT

t.LN.LG.ADT[1] <- 1 - t.LN.LG.ADT[2]

t.LN.LG.AS <- numeric()

t.LN.LG.AS[2] <- P.LN2D.LG.AS

t.LN.LG.AS[1] <- 1 - t.LN.LG.AS[2]

t.LN.LG.M <- numeric()

t.LN.LG.M[2] <- P.LN2D.LG.M

t.LN.LG.M[1] <- 1 - t.LN.LG.M[2]

t.Dead <- c(0,1)

ages <- c(50 : (50 + n.t - 1))

Tr.NoPCa<-array(0, dim = c(2, 2, n.t),

dimnames = list(state_names, state_names, ages))

Tr.NoPCa["Long term", "Dead", ] <- p_mor_age_NoPCa

Tr.NoPCa["Long term", "Long term", ]<-1-p_mor_age_NoPCa

Tr.NoPCa["Dead", "Dead", ]<-1

####

Tr.LG.M.ar<-Tr.LG.RT.ar<-Tr.LG.RP.ar<-Tr.LG.ADT.ar<-Tr.LG.AS.ar<-Tr.HG.M.ar<-Tr.HG.RT.ar<-Tr.HG.RP.ar<-Tr.HG.ADT.ar<-Tr.HG.WW.ar<-array(0, dim = c(2, 2, n.t),

dimnames = list(state_names, state_names, ages))

Tr.HG.RT.ar["Long term", "Dead", ]<-p_mor_age_HG+ ((1-p_mor_age_HG)*t.LN.HG.RT[2])

Tr.HG.RT.ar["Long term", "Long term", ]<-1-Tr.HG.RT.ar["Long term", "Dead", ]

Tr.HG.RT.ar["Dead", "Dead", ]<-1

Tr.HG.RP.ar["Long term", "Dead", ]<-p_mor_age_HG+ ((1-p_mor_age_HG)*t.LN.HG.RP[2])

Tr.HG.RP.ar["Long term", "Long term", ]<-1-Tr.HG.RP.ar["Long term", "Dead", ]

Tr.HG.RP.ar["Dead", "Dead", ]<-1

Tr.HG.ADT.ar["Long term", "Dead", ]<-p_mor_age_HG+ ((1-p_mor_age_HG)*t.LN.HG.ADT[2])

Tr.HG.ADT.ar["Long term", "Long term", ]<-1-Tr.HG.ADT.ar["Long term", "Dead", ]

Tr.HG.ADT.ar["Dead", "Dead", ]<-1

Tr.HG.WW.ar["Long term", "Dead", ]<-p_mor_age_HG+ ((1-p_mor_age_HG)*t.LN.HG.WW[2])

Tr.HG.WW.ar["Long term", "Long term", ]<-1-Tr.HG.WW.ar["Long term", "Dead", ]

Tr.HG.WW.ar["Dead", "Dead", ]<-1

Tr.HG.M.ar["Long term", "Dead", ]<-p_mor_age_HG+ ((1-p_mor_age_HG)*t.LN.HG.M[2])

Tr.HG.M.ar["Long term", "Long term", ]<-1-Tr.HG.M.ar["Long term", "Dead", ]

Tr.HG.M.ar["Dead", "Dead", ]<-1

Tr.LG.RT.ar["Long term", "Dead", ]<-p_mor_age_LG+ ((1-p_mor_age_LG)*t.LN.LG.RT[2])

Tr.LG.RT.ar["Long term", "Long term", ]<-1-Tr.LG.RT.ar["Long term", "Dead", ]

Tr.LG.RT.ar["Dead", "Dead", ]<-1

Tr.LG.RP.ar["Long term", "Dead", ]<-p_mor_age_LG+ ((1-p_mor_age_LG)*t.LN.LG.RP[2])

Tr.LG.RP.ar["Long term", "Long term", ]<-1-Tr.LG.RP.ar["Long term", "Dead", ]

Tr.LG.RP.ar["Dead", "Dead", ]<-1

Tr.LG.ADT.ar["Long term", "Dead", ]<-p_mor_age_LG+ ((1-p_mor_age_LG)*t.LN.LG.ADT[2])

Tr.LG.ADT.ar["Long term", "Long term", ]<-1-Tr.LG.ADT.ar["Long term", "Dead", ]

Tr.LG.ADT.ar["Dead", "Dead", ]<-1

Tr.LG.AS.ar["Long term", "Dead", ]<-p_mor_age_LG+ ((1-p_mor_age_LG)*t.LN.LG.AS[2])

Tr.LG.AS.ar["Long term", "Long term", ]<-1-Tr.LG.AS.ar["Long term", "Dead", ]

Tr.LG.AS.ar["Dead", "Dead", ]<-1

Tr.LG.M.ar["Long term", "Dead", ]<-p_mor_age_LG+ ((1-p_mor_age_LG)*t.LN.LG.M[2])

Tr.LG.M.ar["Long term", "Long term", ]<-1-Tr.LG.M.ar["Long term", "Dead", ]

Tr.LG.M.ar["Dead", "Dead", ]<-1

####

CTr.D.RP.HG<-c(CLN.HG.RP,CDead)

names(CTr.D.RP.HG) <- state_names

CTr.D.RT.HG<-c(CLN.HG.RT,CDead)

names(CTr.D.RT.HG) <- state_names

CTr.D.ADT.HG<-c(CLN.HG.ADT,CDead)

names(CTr.D.ADT.HG) <- state_names

CTr.D.AS.HG<-c(CLN.HG.AS,CDead)

names(CTr.D.AS.HG) <- state_names

CTr.D.MD.HG<-c(CLN.HG.MD,CDead)

names(CTr.D.MD.HG) <- state_names

CTr.D.NoPCa<-c(0,CDead)

names(CTr.D.NoPCa) <- state_names

####

CTr.D.RP.LG<-c(CLN.LG.RP,CDead)

names(CTr.D.RP.LG) <- state_names

CTr.D.RT.LG<-c(CLN.LG.RT,CDead)

names(CTr.D.RT.LG) <- state_names

CTr.D.ADT.LG<-c(CLN.LG.ADT,CDead)

names(CTr.D.ADT.LG) <- state_names

CTr.D.AS.LG<-c(CLN.LG.AS,CDead)

names(CTr.D.AS.LG) <- state_names

CTr.D.MD.LG<-c(CLN.LG.MD,CDead)

names(CTr.D.MD.LG) <- state_names

dAd<-(dULN.PaT*30/216)+(dULN.AS*186/216)

dULN.HG.MD<-( dAd*0.225)+( dULN.AS*(1-0.225))

dULN.LG.MD<-( dAd*0.048)+( dULN.AS*(1-0.048))

ULN.RP<-1-dULN.RP

ULN.RT<-1-dULN.RT

ULN.AS<-1-(( dAd*0.007)+( dULN.AS*(1-0.007)))

ULN.WW<-1-(( dAd*0.049)+( dULN.AS*(1-0.049)))

ULN.ADT<-1-dULN.ADT

ULN.HG.MD<-1-dULN.HG.MD

ULN.LG.MD<-1-dULN.LG.MD

UDead <- 0

UTr.D.RP<-c(ULN.RP,UDead)

names(UTr.D.RP) <- state_names

UTr.D.RT<-c(ULN.RT,UDead)

names(UTr.D.RT) <- state_names

UTr.D.ADT<-c(ULN.ADT,UDead)

names(UTr.D.ADT) <- state_names

UTr.D.AS<-c(ULN.AS,UDead)

names(UTr.D.AS) <- state_names

UTr.D.WW<-c(ULN.WW,UDead)

names(UTr.D.WW) <- state_names

UTr.HG.MD<-c(ULN.HG.MD,UDead)

#UTr.HG.MD<-c(ULN.AS,UDead)

names(UTr.HG.MD) <- state_names

UTr.LG.MD<-c(ULN.LG.MD,UDead)

#UTr.LG.MD<-c(ULN.AS,UDead)

names(UTr.LG.MD) <- state_names

UTr.D.NoPCa<-c(1,UDead)

names(UTr.D.NoPCa) <- state_names

#####################################################################

MX.SOC<-MX.SOC.HG.RP<-MX.SOC.HG.RT<-MX.SOC.HG.WW <-MX.SOC.HG.ADT<-

MX.SOC.LG.AS<-MX.SOC.LG.RP<-MX.SOC.LG.RT<-MX.SOC.LG.ADT<-

matrix(0, ncol = 2, nrow = n.t + 1, dimnames = list(c(50 : (50 + n.t)), state_names))

#initiate first cycle

MX.SOC.HG.RP[1,]<-c(1*p.PCa*p.HG*p.HG.RP,0)

MX.SOC.HG.RT[1,]<-c(1*p.PCa*p.HG*p.HG.RT,0)

MX.SOC.HG.WW[1,]<-c(1*p.PCa*p.HG*p.HG.WW,0)

MX.SOC.HG.ADT[1,]<-c(1*p.PCa*p.HG*p.HG.ADT,0)

MX.SOC.LG.RP[1,]<-c(1*p.PCa*p.LG*p.LG.RP,0)

MX.SOC.LG.RT[1,]<-c(1*p.PCa*p.LG*p.LG.RT,0)

MX.SOC.LG.AS[1,]<-c(1*p.PCa*p.LG*p.LG.AS,0)

MX.SOC.LG.ADT[1,]<-c(1*p.PCa*p.LG*p.LG.ADT,0)

for (t in 1:n.t) {

MX.SOC.HG.RP[t+1,]<-MX.SOC.HG.RP[t,]%*%Tr.HG.RP.ar[, , t]

MX.SOC.HG.RT[t+1,]<-MX.SOC.HG.RT[t,]%*%Tr.HG.RT.ar[, , t]

MX.SOC.HG.WW[t+1,]<-MX.SOC.HG.WW[t,]%*%Tr.HG.WW.ar[, , t]

MX.SOC.HG.ADT[t+1,]<-MX.SOC.HG.ADT[t,]%*%Tr.HG.ADT.ar[, , t]

MX.SOC.LG.RP[t+1,]<-MX.SOC.LG.RP[t,]%*%Tr.LG.RP.ar[, , t]

MX.SOC.LG.RT[t+1,]<-MX.SOC.LG.RT[t,]%*%Tr.LG.RT.ar[, , t]

MX.SOC.LG.AS[t+1,]<-MX.SOC.LG.AS[t,]%*%Tr.LG.AS.ar[, , t]

MX.SOC.LG.ADT[t+1,]<-MX.SOC.LG.ADT[t,]%*%Tr.LG.ADT.ar[, , t]

}

MX.SOCall<-MX.SOC.HG.RP+MX.SOC.HG.RT+MX.SOC.HG.WW+MX.SOC.HG.ADT+

MX.SOC.LG.RP+MX.SOC.LG.RT+MX.SOC.LG.AS+MX.SOC.LG.ADT

MX.SOC.LG<-MX.SOC.LG.RP+MX.SOC.LG.RT+MX.SOC.LG.AS

MX.SOC.NoPCa <- matrix(0, ncol = 2, nrow = n.t + 1,

dimnames = list(c(50 : (50 + n.t)), state_names))

MX.SOC.NoPCa[1,]<-c(1-p.PCa,0)

for(t in 1 : n.t){

MX.SOC.NoPCa[t + 1, ] <- MX.SOC.NoPCa[t, ] %*% Tr.NoPCa[, , t]

}

###################################################################

meanc.SOC.HG.RP<- MX.SOC.HG.RP%*%CTr.D.RP.HG

meanc.SOC.HG.RP[1,1]<-(CRP.Y1.HG)*MX.SOC.HG.RP[1,1]

meanc.SOC.HG.RP[2,1]<-(CRP.Y2.HG)*MX.SOC.HG.RP[2,1]

DCost.SOC.HG.RP<-Dead.SOC.HG.RP<- matrix(0, ncol = 1, nrow = n.t + 1,

dimnames = list(c(50 : (50 + n.t)), "DEAD"))

for(t in 1 : n.t+1){

Dead.SOC.HG.RP[t] <- MX.SOC.HG.RP[t,2 ]-MX.SOC.HG.RP[t-1,2 ]

}

DCost.SOC.HG.RP[1:2]<-(Cdea.Y-CRP.Y2.HG)*Dead.SOC.HG.RP[1:2]

DCost.SOC.HG.RP[3:n.t+1]<-(Cdea.Y-CLN.HG.RP)*Dead.SOC.HG.RP[3:n.t+1]

mean.SOC.HG.RP<-meanc.SOC.HG.RP+DCost.SOC.HG.RP

####

meanc.SOC.HG.RT<- MX.SOC.HG.RT%*%CTr.D.RT.HG

meanc.SOC.HG.RT[1,1]<-(CRT.Y1.HG)*MX.SOC.HG.RT[1,1]

meanc.SOC.HG.RT[2,1]<-(CRT.Y2.HG)*MX.SOC.HG.RT[2,1]

DCost.SOC.HG.RT<-Dead.SOC.HG.RT<- matrix(0, ncol = 1, nrow = n.t + 1,

dimnames = list(c(50 : (50 + n.t)), "DEAD"))

for(t in 1 : n.t+1){

Dead.SOC.HG.RT[t] <- MX.SOC.HG.RT[t,2 ]-MX.SOC.HG.RT[t-1,2 ]

}

DCost.SOC.HG.RT[1:2]<-(Cdea.Y-CRT.Y2.HG)*Dead.SOC.HG.RT[1:2]

DCost.SOC.HG.RT[3:n.t+1]<-(Cdea.Y-CLN.HG.RT)*Dead.SOC.HG.RT[3:n.t+1]

mean.SOC.HG.RT<-meanc.SOC.HG.RT+DCost.SOC.HG.RT

####

meanc.SOC.HG.WW<- MX.SOC.HG.WW%*%CTr.D.AS.HG

meanc.SOC.HG.WW[1,1]<-(CAS.Y1.HG)*MX.SOC.HG.WW[1,1]

meanc.SOC.HG.WW[2,1]<-(CAS.Y2.HG)*MX.SOC.HG.WW[2,1]

DCost.SOC.HG.WW<-Dead.SOC.HG.WW<- matrix(0, ncol = 1, nrow = n.t + 1,

dimnames = list(c(50 : (50 + n.t)), "DEAD"))

for(t in 1 : n.t+1){

Dead.SOC.HG.WW[t] <- MX.SOC.HG.WW[t,2 ]-MX.SOC.HG.WW[t-1,2 ]

}

DCost.SOC.HG.WW[1:2]<-(Cdea.Y-CAS.Y2.HG)*Dead.SOC.HG.WW[1:2]

DCost.SOC.HG.WW[3:n.t+1]<-(Cdea.Y-CLN.HG.AS)*Dead.SOC.HG.WW[3:n.t+1]

mean.SOC.HG.WW<-meanc.SOC.HG.WW+DCost.SOC.HG.WW

####

meanc.SOC.HG.ADT<- MX.SOC.HG.ADT%*%CTr.D.ADT.HG

meanc.SOC.HG.ADT[1,1]<-(CADT.Y1.HG)*MX.SOC.HG.ADT[1,1]

meanc.SOC.HG.ADT[2,1]<-(CADT.Y2.HG)*MX.SOC.HG.ADT[2,1]

DCost.SOC.HG.ADT<-Dead.SOC.HG.ADT<- matrix(0, ncol = 1, nrow = n.t + 1,

dimnames = list(c(50 : (50 + n.t)), "DEAD"))

for(t in 1 : n.t+1){

Dead.SOC.HG.ADT[t] <- MX.SOC.HG.ADT[t,2 ]-MX.SOC.HG.ADT[t-1,2 ]

}

DCost.SOC.HG.ADT[1:2]<-(Cdea.Y-CADT.Y2.HG)*Dead.SOC.HG.ADT[1:2]

DCost.SOC.HG.ADT[3:n.t+1]<-(Cdea.Y-CLN.HG.ADT)*Dead.SOC.HG.ADT[3:n.t+1]

mean.SOC.HG.ADT<-meanc.SOC.HG.ADT+DCost.SOC.HG.ADT

meanc.SOC.LG.RP<- MX.SOC.LG.RP%*%CTr.D.RP.LG

meanc.SOC.LG.RP[1,1]<-(CRP.Y1.LG)*MX.SOC.LG.RP[1,1]

meanc.SOC.LG.RP[2,1]<-(CRP.Y2.LG)*MX.SOC.LG.RP[2,1]

DCost.SOC.LG.RP<-Dead.SOC.LG.RP<- matrix(0, ncol = 1, nrow = n.t + 1,

dimnames = list(c(50 : (50 + n.t)), "DEAD"))

for(t in 1 : n.t+1){

Dead.SOC.LG.RP[t] <- MX.SOC.LG.RP[t,2 ]-MX.SOC.LG.RP[t-1,2 ]

}

DCost.SOC.LG.RP[1:2]<-(Cdea.Y-CRP.Y2.LG)*Dead.SOC.LG.RP[1:2]

DCost.SOC.LG.RP[3:n.t+1]<-(Cdea.Y-CLN.LG.RP)*Dead.SOC.LG.RP[3:n.t+1]

mean.SOC.LG.RP<-meanc.SOC.LG.RP+DCost.SOC.LG.RP

####

meanc.SOC.LG.RT<- MX.SOC.LG.RT%*%CTr.D.RT.LG

meanc.SOC.LG.RT[1,1]<-(CRT.Y1.LG)*MX.SOC.LG.RT[1,1]

meanc.SOC.LG.RT[2,1]<-(CRT.Y2.LG)*MX.SOC.LG.RT[2,1]

DCost.SOC.LG.RT<-Dead.SOC.LG.RT<- matrix(0, ncol = 1, nrow = n.t + 1,

dimnames = list(c(50 : (50 + n.t)), "DEAD"))

for(t in 1 : n.t+1){

Dead.SOC.LG.RT[t] <- MX.SOC.LG.RT[t,2 ]-MX.SOC.LG.RT[t-1,2 ]

}

DCost.SOC.LG.RT[1:2]<-(Cdea.Y-CRT.Y2.LG)*Dead.SOC.LG.RT[1:2]

DCost.SOC.LG.RT[3:n.t+1]<-(Cdea.Y-CLN.LG.RT)*Dead.SOC.LG.RT[3:n.t+1]

mean.SOC.LG.RT<-meanc.SOC.LG.RT+DCost.SOC.LG.RT

####

meanc.SOC.LG.AS<- MX.SOC.LG.AS%*%CTr.D.AS.LG

meanc.SOC.LG.AS[1,1]<-(CAS.Y1.LG)*MX.SOC.LG.AS[1,1]

meanc.SOC.LG.AS[2,1]<-(CAS.Y2.LG)*MX.SOC.LG.AS[2,1]

DCost.SOC.LG.AS<-Dead.SOC.LG.AS<- matrix(0, ncol = 1, nrow = n.t + 1,

dimnames = list(c(50 : (50 + n.t)), "DEAD"))

for(t in 1 : n.t+1){

Dead.SOC.LG.AS[t] <- MX.SOC.LG.AS[t,2 ]-MX.SOC.LG.AS[t-1,2 ]

}

DCost.SOC.LG.AS[1:2]<-(Cdea.Y-CAS.Y2.LG)*Dead.SOC.LG.AS[1:2]

DCost.SOC.LG.AS[3:n.t+1]<-(Cdea.Y-CLN.LG.AS)*Dead.SOC.LG.AS[3:n.t+1]

mean.SOC.LG.AS<-meanc.SOC.LG.RT+DCost.SOC.LG.AS

####

meanc.SOC.LG.ADT<- MX.SOC.LG.ADT%*%CTr.D.ADT.LG

meanc.SOC.LG.ADT[1,1]<-(CADT.Y1.LG)*MX.SOC.LG.ADT[1,1]

meanc.SOC.LG.ADT[2,1]<-(CADT.Y2.LG)*MX.SOC.LG.ADT[2,1]

DCost.SOC.LG.ADT<-Dead.SOC.LG.ADT<- matrix(0, ncol = 1, nrow = n.t + 1,

dimnames = list(c(50 : (50 + n.t)), "DEAD"))

for(t in 1 : n.t+1){

Dead.SOC.LG.ADT[t] <- MX.SOC.LG.ADT[t,2 ]-MX.SOC.LG.ADT[t-1,2 ]

}

DCost.SOC.LG.ADT[1:2]<-(Cdea.Y-CADT.Y2.LG)*Dead.SOC.LG.ADT[1:2]

DCost.SOC.LG.ADT[3:n.t+1]<-(Cdea.Y-CLN.LG.ADT)*Dead.SOC.LG.ADT[3:n.t+1]

mean.SOC.LG.ADT<-meanc.SOC.LG.ADT+DCost.SOC.LG.ADT

mean.SOC.NoPCa<- MX.SOC.NoPCa%*%CTr.D.NoPCa

mean.SOC.NoPCa[1,1]<-(CBiopsy)*MX.SOC.NoPCa[1,1]

meanc.SOC<-mean.SOC.HG.RP+mean.SOC.HG.RT+mean.SOC.HG.WW+mean.SOC.HG.ADT+

mean.SOC.LG.RP+mean.SOC.LG.RT+mean.SOC.LG.AS+mean.SOC.LG.ADT+mean.SOC.NoPCa

rDiscount.U <- 0.015 #CADTH recommendation

rDiscount.C <- 0.015 #

cycle_rDiscount.U <- 1/(1 + rDiscount.U)^(0:n.t)

cycle_rDiscount.C <- 1/(1 + rDiscount.C)^(0:n.t)

DisC.SOC.popdata<- t(meanc.SOC) %*% cycle_rDiscount.C

meanu.SOC.HG.RP<- MX.SOC.HG.RP%*%UTr.D.RP

meanu.SOC.HG.RP[1,1]<-(1-dUPCa-dUBiopsy-dURP)*MX.SOC.HG.RP[1,1]

meanu.SOC.HG.RT<- MX.SOC.HG.RT%*%UTr.D.RT

meanu.SOC.HG.RT[1,1]<-(1-dUPCa-dUBiopsy-dURT)*MX.SOC.HG.RT[1,1]

meanu.SOC.HG.WW<- MX.SOC.HG.WW%*%UTr.D.WW

meanu.SOC.HG.WW[1,1]<-(1-dUBiopsy-dUPCa)*MX.SOC.HG.WW[1,1]

meanu.SOC.HG.ADT<- MX.SOC.HG.ADT%*%UTr.D.ADT

meanu.SOC.HG.ADT[1,1]<-(1-dUBiopsy-dUPCa-dUADT)*MX.SOC.HG.ADT[1,1]

meanu.SOC.LG.RP<- MX.SOC.LG.RP%*%UTr.D.RP

meanu.SOC.LG.RP[1,1]<-(1-dUPCa-dUBiopsy-dURP)*MX.SOC.LG.RP[1,1]

meanu.SOC.LG.RT<- MX.SOC.HG.RT%*%UTr.D.RT

meanu.SOC.LG.RT[1,1]<-(1-dUPCa-dUBiopsy-dURT)*MX.SOC.LG.RT[1,1]

meanu.SOC.LG.AS<- MX.SOC.LG.AS%*%UTr.D.AS

meanu.SOC.LG.AS[1,1]<-(1-dUBiopsy-dUPCa)*MX.SOC.LG.AS[1,1]

meanu.SOC.LG.ADT<- MX.SOC.LG.ADT%*%UTr.D.ADT#

meanu.SOC.LG.ADT[1,1]<-(1-dUBiopsy-dUPCa-dUADT)*MX.SOC.LG.ADT[1,1]

meanu.SOC.NoPCa<- MX.SOC.NoPCa%*%UTr.D.NoPCa#

meanu.SOC.NoPCa[1,1]<-(1-dUBiopsy)*MX.SOC.NoPCa[1,1]

meanu.SOC<-meanu.SOC.HG.RP+meanu.SOC.HG.RT+meanu.SOC.HG.WW+meanu.SOC.HG.ADT+

meanu.SOC.LG.RP+meanu.SOC.LG.RT+meanu.SOC.LG.AS+meanu.SOC.LG.ADT+meanu.SOC.NoPCa

DisU.SOC.popdata<- t(meanu.SOC) %*% cycle_rDiscount.U

#############################

#####################################################################

MX.BIO<-MX.BIO.HG.RP<-MX.BIO.HG.RT<-MX.BIO.HG.WW <-MX.BIO.HG.ADT<-

MX.BIO.LG.AS<-MX.BIO.LG.RP<-MX.BIO.LG.RT<-MX.BIO.LG.ADT<-MX.BIO.HG.MD<-MX.BIO.LG.MD<-

matrix(0, ncol = 2, nrow = n.t + 1, dimnames = list(c(50 : (50 + n.t)), state_names))

MX.BIO.HG.RP[1,]<-c(1*p.PCa*p.HG*Bio.Sens.HG*p.HG.RP,0)

MX.BIO.HG.RT[1,]<-c(1*p.PCa*p.HG*Bio.Sens.HG*p.HG.RT,0)

MX.BIO.HG.WW[1,]<-c(1*p.PCa*p.HG*Bio.Sens.HG*p.HG.WW,0)

MX.BIO.HG.ADT[1,]<-c(1*p.PCa*p.HG*Bio.Sens.HG*p.HG.ADT,0)

MX.BIO.HG.MD[1,]<-c(1*p.PCa*p.HG*(1-Bio.Sens.HG),0) ##Missed HG

hg<-MX.BIO.HG.RP[1,]+MX.BIO.HG.RT[1,]+MX.BIO.HG.WW[1,]+MX.BIO.HG.ADT[1,]+MX.BIO.HG.MD[1,]

MX.BIO.LG.RP[1,]<-c(1*p.PCa*p.LG*(1-Bio.Spec.LG)*p.LG.RP,0)

MX.BIO.LG.RT[1,]<-c(1*p.PCa*p.LG*(1-Bio.Spec.LG)*p.LG.RT,0)

MX.BIO.LG.AS[1,]<-c(1*p.PCa*p.LG*(1-Bio.Spec.LG)*p.LG.AS,0)

MX.BIO.LG.ADT[1,]<-c(1*p.PCa*p.LG*(1-Bio.Spec.LG)*p.LG.ADT,0)

MX.BIO.LG.MD[1,]<-c(1*p.PCa*p.LG*Bio.Spec.LG,0)#Missed LG

Lg<-MX.BIO.LG.RP[1,]+MX.BIO.LG.RT[1,]+MX.BIO.LG.AS[1,]+MX.BIO.LG.ADT[1,]+MX.BIO.LG.MD[1,]

for (t in 1:n.t) {

MX.BIO.HG.RP[t+1,]<-MX.BIO.HG.RP[t,]%*%Tr.HG.RP.ar[, , t]

MX.BIO.HG.RT[t+1,]<-MX.BIO.HG.RT[t,]%*%Tr.HG.RT.ar[, , t]

MX.BIO.HG.WW[t+1,]<-MX.BIO.HG.WW[t,]%*%Tr.HG.WW.ar[, , t]

MX.BIO.HG.ADT[t+1,]<-MX.BIO.HG.ADT[t,]%*%Tr.HG.ADT.ar[, , t]

MX.BIO.HG.MD[t+1,]<-MX.BIO.HG.MD[t,]%*%Tr.HG.M.ar[, , t]

MX.BIO.LG.RP[t+1,]<-MX.BIO.LG.RP[t,]%*%Tr.LG.RP.ar[, , t]

MX.BIO.LG.RT[t+1,]<-MX.BIO.LG.RT[t,]%*%Tr.LG.RT.ar[, , t]

MX.BIO.LG.AS[t+1,]<-MX.BIO.LG.AS[t,]%*%Tr.LG.AS.ar[, , t]

MX.BIO.LG.ADT[t+1,]<-MX.BIO.LG.ADT[t,]%*%Tr.LG.ADT.ar[, , t]

MX.BIO.LG.MD[t+1,]<-MX.BIO.LG.MD[t,]%*%Tr.LG.M.ar[, , t]

}

MX.BIOall<-MX.BIO.HG.RP+MX.BIO.HG.RT+MX.BIO.HG.WW+MX.BIO.HG.ADT+

MX.BIO.LG.RP+MX.BIO.LG.RT+MX.BIO.LG.AS+MX.BIO.LG.ADT+MX.BIO.LG.MD+MX.BIO.HG.MD

MX.BIO.LG<-MX.BIO.LG.RP+MX.BIO.LG.RT+MX.BIO.LG.AS+MX.BIO.LG.ADT+MX.BIO.LG.MD

MX.BIO.HG<-MX.BIO.HG.RP+MX.BIO.HG.RT+MX.BIO.HG.WW+MX.BIO.HG.ADT+MX.BIO.HG.MD

#####################

MX.BIO.NoPCa.P <- matrix(0, ncol = 2, nrow = n.t + 1,

dimnames = list(c(50 : (50 + n.t)), state_names))

MX.BIO.NoPCa.P[1,]<-c((1-p.PCa)*(1-Bio.Spec.NoPca),0)

MX.BIO.NoPCa.N <- matrix(0, ncol = 2, nrow = n.t + 1,

dimnames = list(c(50 : (50 + n.t)), state_names))

MX.BIO.NoPCa.N[1,]<-c((1-p.PCa)*(Bio.Spec.NoPca),0)

for(t in 1 : n.t){

MX.BIO.NoPCa.P[t + 1, ] <- MX.BIO.NoPCa.P[t, ] %*% Tr.NoPCa[, , t]

MX.BIO.NoPCa.N[t + 1, ] <- MX.BIO.NoPCa.N[t, ] %*% Tr.NoPCa[, , t]

}

MX.BIO.NoPCa<-MX.BIO.NoPCa.P+MX.BIO.NoPCa.N

###################################################################

meanc.BIO.HG.RP<- MX.BIO.HG.RP%*%CTr.D.RP.HG

meanc.BIO.HG.RP[1,1]<-(CRP.Y1.HG+CBiomarker)*MX.BIO.HG.RP[1,1]

meanc.BIO.HG.RP[2,1]<-(CRP.Y2.HG)*MX.BIO.HG.RP[2,1]

DCost.BIO.HG.RP<-Dead.BIO.HG.RP<- matrix(0, ncol = 1, nrow = n.t + 1,

dimnames = list(c(50 : (50 + n.t)), "DEAD"))

for(t in 1 : n.t+1){

Dead.BIO.HG.RP[t] <- MX.BIO.HG.RP[t,2 ]-MX.BIO.HG.RP[t-1,2 ]

}

DCost.BIO.HG.RP[1:2]<-(Cdea.Y-CRP.Y2.HG)*Dead.BIO.HG.RP[1:2]

DCost.BIO.HG.RP[3:n.t+1]<-(Cdea.Y-CLN.HG.RP)*Dead.BIO.HG.RP[3:n.t+1]

meanct.BIO.HG.RP<-meanc.BIO.HG.RP+DCost.BIO.HG.RP

####

meanc.BIO.HG.RT<- MX.BIO.HG.RT%*%CTr.D.RT.HG

meanc.BIO.HG.RT[1,1]<-(CRT.Y1.HG+CBiomarker)*MX.BIO.HG.RT[1,1]

meanc.BIO.HG.RT[2,1]<-(CRT.Y2.HG)*MX.BIO.HG.RT[2,1]

DCost.BIO.HG.RT<-Dead.BIO.HG.RT<- matrix(0, ncol = 1, nrow = n.t + 1,

dimnames = list(c(50 : (50 + n.t)), "DEAD"))

for(t in 1 : n.t+1){

Dead.BIO.HG.RT[t] <- MX.BIO.HG.RT[t,2 ]-MX.BIO.HG.RT[t-1,2 ]

}

DCost.BIO.HG.RT[1:2]<-(Cdea.Y-CRT.Y2.HG)*Dead.BIO.HG.RT[1:2]

DCost.BIO.HG.RT[3:n.t+1]<-(Cdea.Y-CLN.HG.RT)*Dead.BIO.HG.RT[3:n.t+1]

meanct.BIO.HG.RT<-meanc.BIO.HG.RT+DCost.BIO.HG.RT

####

meanc.BIO.HG.WW<- MX.BIO.HG.WW%*%CTr.D.AS.HG#

meanc.BIO.HG.WW[1,1]<-(CAS.Y1.HG+CBiomarker)*MX.BIO.HG.WW[1,1]#

meanc.BIO.HG.WW[2,1]<-(CAS.Y2.HG)*MX.BIO.HG.WW[2,1]#

DCost.BIO.HG.WW<-Dead.BIO.HG.WW<- matrix(0, ncol = 1, nrow = n.t + 1,

dimnames = list(c(50 : (50 + n.t)), "DEAD"))

for(t in 1 : n.t+1){

Dead.BIO.HG.WW[t] <- MX.BIO.HG.WW[t,2 ]-MX.BIO.HG.WW[t-1,2 ]

}

DCost.BIO.HG.WW[1:2]<-(Cdea.Y-CAS.Y2.HG)*Dead.BIO.HG.WW[1:2]

DCost.BIO.HG.WW[3:n.t+1]<-(Cdea.Y-CLN.HG.AS)*Dead.BIO.HG.WW[3:n.t+1]

meanct.BIO.HG.WW<-meanc.BIO.HG.WW+DCost.BIO.HG.WW

####

meanc.BIO.HG.ADT<- MX.BIO.HG.ADT%*%CTr.D.ADT.HG

meanc.BIO.HG.ADT[1,1]<-(CADT.Y1.HG+CBiomarker)*MX.BIO.HG.ADT[1,1]

meanc.BIO.HG.ADT[2,1]<-(CADT.Y2.HG)*MX.BIO.HG.ADT[2,1]

DCost.BIO.HG.ADT<-Dead.BIO.HG.ADT<- matrix(0, ncol = 1, nrow = n.t + 1,

dimnames = list(c(50 : (50 + n.t)), "DEAD"))

for(t in 1 : n.t+1){

Dead.BIO.HG.ADT[t] <- MX.BIO.HG.ADT[t,2 ]-MX.BIO.HG.ADT[t-1,2 ]

}

DCost.BIO.HG.ADT[1:2]<-(Cdea.Y-CADT.Y2.HG)*Dead.BIO.HG.ADT[1:2]

DCost.BIO.HG.ADT[3:n.t+1]<-(Cdea.Y-CLN.HG.ADT)*Dead.BIO.HG.ADT[3:n.t+1]

meanct.BIO.HG.ADT<-meanc.BIO.HG.ADT+DCost.BIO.HG.ADT

meanc.BIO.HG.MD<- MX.BIO.HG.MD%*%CTr.D.MD.HG

meanc.BIO.HG.MD[1,1]<-(CMD.Y1.HG+CBiomarker)*MX.BIO.HG.MD[1,1]

meanc.BIO.HG.MD[2,1]<-(CMD.Y2.HG)*MX.BIO.HG.MD[2,1]

DCost.BIO.HG.MD<-Dead.BIO.HG.MD<- matrix(0, ncol = 1, nrow = n.t + 1,

dimnames = list(c(50 : (50 + n.t)), "DEAD"))

for(t in 1 : n.t+1){

Dead.BIO.HG.MD[t] <- MX.BIO.HG.MD[t,2 ]-MX.BIO.HG.MD[t-1,2 ]

}

DCost.BIO.HG.MD[1:2]<-(Cdea.Y-CMD.Y2.HG)*Dead.BIO.HG.MD[1:2]

DCost.BIO.HG.MD[3:n.t+1]<-(Cdea.Y-CLN.HG.MD)*Dead.BIO.HG.MD[3:n.t+1]

meanct.BIO.HG.MD<-meanc.BIO.HG.MD+DCost.BIO.HG.MD

meanc.BIO.LG.RP<- MX.BIO.LG.RP%*%CTr.D.RP.LG

meanc.BIO.LG.RP[1,1]<-(CRP.Y1.LG+CBiomarker)*MX.BIO.LG.RP[1,1]

meanc.BIO.LG.RP[2,1]<-(CRP.Y2.LG)*MX.BIO.LG.RP[2,1]

DCost.BIO.LG.RP<-Dead.BIO.LG.RP<- matrix(0, ncol = 1, nrow = n.t + 1,

dimnames = list(c(50 : (50 + n.t)), "DEAD"))

for(t in 1 : n.t+1){

Dead.BIO.LG.RP[t] <- MX.BIO.LG.RP[t,2 ]-MX.BIO.LG.RP[t-1,2 ]

}

DCost.BIO.LG.RP[1:2]<-(Cdea.Y-CRP.Y2.LG)*Dead.BIO.LG.RP[1:2]

DCost.BIO.LG.RP[3:n.t+1]<-(Cdea.Y-CLN.LG.RP)*Dead.BIO.LG.RP[3:n.t+1]

meanct.BIO.LG.RP<-meanc.BIO.LG.RP+DCost.BIO.LG.RP

####

meanc.BIO.LG.RT<- MX.BIO.LG.RT%*%CTr.D.RT.LG

meanc.BIO.LG.RT[1,1]<-(CRT.Y1.LG+CBiomarker)*MX.BIO.LG.RT[1,1]

meanc.BIO.LG.RT[2,1]<-(CRT.Y2.LG)*MX.BIO.LG.RT[2,1]

DCost.BIO.LG.RT<-Dead.BIO.LG.RT<- matrix(0, ncol = 1, nrow = n.t + 1,

dimnames = list(c(50 : (50 + n.t)), "DEAD"))

for(t in 1 : n.t+1){

Dead.BIO.LG.RT[t] <- MX.BIO.LG.RT[t,2 ]-MX.BIO.LG.RT[t-1,2 ]

}

DCost.BIO.LG.RT[1:2]<-(Cdea.Y-CRT.Y2.LG)*Dead.BIO.LG.RT[1:2]

DCost.BIO.LG.RT[3:n.t+1]<-(Cdea.Y-CLN.LG.RT)*Dead.BIO.LG.RT[3:n.t+1]

meanct.BIO.LG.RT<-meanc.BIO.LG.RT+DCost.BIO.LG.RT

####

meanc.BIO.LG.AS<- MX.BIO.LG.AS%*%CTr.D.AS.LG

meanc.BIO.LG.AS[1,1]<-(CAS.Y1.LG+CBiomarker)*MX.BIO.LG.AS[1,1]

meanc.BIO.LG.AS[2,1]<-(CAS.Y2.LG)*MX.BIO.LG.AS[2,1]

DCost.BIO.LG.AS<-Dead.BIO.LG.AS<- matrix(0, ncol = 1, nrow = n.t + 1,

dimnames = list(c(50 : (50 + n.t)), "DEAD"))

for(t in 1 : n.t+1){

Dead.BIO.LG.AS[t] <- MX.BIO.LG.AS[t,2 ]-MX.BIO.LG.AS[t-1,2 ]

}

DCost.BIO.LG.AS[1:2]<-(Cdea.Y-CAS.Y2.LG)*Dead.BIO.LG.AS[1:2]

DCost.BIO.LG.AS[3:n.t+1]<-(Cdea.Y-CLN.LG.AS)*Dead.BIO.LG.AS[3:n.t+1]

meanct.BIO.LG.AS<-meanc.BIO.LG.RT+DCost.BIO.LG.AS

####

meanc.BIO.LG.ADT<- MX.BIO.LG.ADT%*%CTr.D.ADT.LG

meanc.BIO.LG.ADT[1,1]<-(CADT.Y1.LG+CBiomarker)*MX.BIO.LG.ADT[1,1]

meanc.BIO.LG.ADT[2,1]<-(CADT.Y2.LG)*MX.BIO.LG.ADT[2,1]

DCost.BIO.LG.ADT<-Dead.BIO.LG.ADT<- matrix(0, ncol = 1, nrow = n.t + 1,

dimnames = list(c(50 : (50 + n.t)), "DEAD"))

for(t in 1 : n.t+1){

Dead.BIO.LG.ADT[t] <- MX.BIO.LG.ADT[t,2 ]-MX.BIO.LG.ADT[t-1,2 ]

}

DCost.BIO.LG.ADT[1:2]<-(Cdea.Y-CADT.Y2.LG)*Dead.BIO.LG.ADT[1:2]

DCost.BIO.LG.ADT[3:n.t+1]<-(Cdea.Y-CLN.LG.ADT)*Dead.BIO.LG.ADT[3:n.t+1]

meanct.BIO.LG.ADT<-meanc.BIO.LG.ADT+DCost.BIO.LG.ADT

####

meanc.BIO.LG.MD<- MX.BIO.LG.MD%*%CTr.D.MD.LG

meanc.BIO.LG.MD[1,1]<-(CMD.Y1.LG+CBiomarker)*MX.BIO.LG.MD[1,1]

meanc.BIO.LG.MD[2,1]<-(CMD.Y2.LG)*MX.BIO.LG.MD[2,1]

DCost.BIO.LG.MD<-Dead.BIO.LG.MD<- matrix(0, ncol = 1, nrow = n.t + 1,

dimnames = list(c(50 : (50 + n.t)), "DEAD"))

for(t in 1 : n.t+1){

Dead.BIO.LG.MD[t] <- MX.BIO.LG.MD[t,2 ]-MX.BIO.LG.MD[t-1,2 ]

}

DCost.BIO.LG.MD[1:2]<-(Cdea.Y-CMD.Y2.LG)*Dead.BIO.LG.MD[1:2]

DCost.BIO.LG.MD[3:n.t+1]<-(Cdea.Y-CLN.LG.MD)*Dead.BIO.LG.MD[3:n.t+1]

meanct.BIO.LG.MD<-meanc.BIO.LG.MD+DCost.BIO.LG.MD

meanc.BIO.NoPCa.p<- MX.BIO.NoPCa.P%*%CTr.D.NoPCa

meanc.BIO.NoPCa.p[1,1]<-(CBiopsy+CBiomarker)*MX.BIO.NoPCa.P[1,1]

meanc.BIO.NoPCa.N<- MX.BIO.NoPCa.N%*%CTr.D.NoPCa

meanc.BIO.NoPCa.N[1,1]<-(CBiomarker)*MX.BIO.NoPCa.N[1,1]

meanc.BIO<-meanct.BIO.HG.RP+meanct.BIO.HG.RT+meanct.BIO.HG.ADT+meanct.BIO.HG.WW+meanct.BIO.HG.MD+

meanct.BIO.LG.RP+meanct.BIO.LG.RT+meanct.BIO.LG.ADT+meanct.BIO.LG.AS+meanct.BIO.LG.MD+meanc.BIO.NoPCa.p+meanc.BIO.NoPCa.N

rDiscount.U <- 0.015

rDiscount.C <- 0.015

cycle_rDiscount.U <- 1/(1 + rDiscount.U)^(0:n.t)

cycle_rDiscount.C <- 1/(1 + rDiscount.C)^(0:n.t)

DisC.BIO.popdata<- t(meanc.BIO) %*% cycle_rDiscount.C

#####Utility

meanu.BIO.HG.RP<- MX.BIO.HG.RP%*%UTr.D.RP

meanu.BIO.HG.RP[1,1]<-(1-dUPCa-dUBiopsy-dURP)*MX.BIO.HG.RP[1,1]

meanu.BIO.HG.RT<- MX.BIO.HG.RT%*%UTr.D.RT

meanu.BIO.HG.RT[1,1]<-(1-dUPCa-dUBiopsy-dURT)*MX.BIO.HG.RT[1,1]

meanu.BIO.HG.WW<- MX.BIO.HG.WW%*%UTr.D.WW

meanu.BIO.HG.WW[1,1]<-(1-dUBiopsy-dUPCa)*MX.BIO.HG.WW[1,1]

meanu.BIO.HG.ADT<- MX.BIO.HG.ADT%*%UTr.D.ADT

meanu.BIO.HG.ADT[1,1]<-(1-dUBiopsy-dUPCa-dUADT)*MX.BIO.HG.ADT[1,1]

meanu.BIO.HG.MD<- MX.BIO.HG.MD%*%UTr.HG.MD

meanu.BIO.HG.MD[1,1]<-(1-dUAS)*MX.BIO.HG.MD[1,1]

meanu.BIO.LG.RP<- MX.BIO.LG.RP%*%UTr.D.RP

meanu.BIO.LG.RP[1,1]<-(1-dUPCa-dUBiopsy-dURP)*MX.BIO.LG.RP[1,1]

meanu.BIO.LG.RT<- MX.BIO.HG.RT%*%UTr.D.RT

meanu.BIO.LG.RT[1,1]<-(1-dUPCa-dUBiopsy-dURT)*MX.BIO.LG.RT[1,1]

meanu.BIO.LG.AS<- MX.BIO.LG.AS%*%UTr.D.AS

meanu.BIO.LG.AS[1,1]<-(1-dUBiopsy-dUPCa)*MX.BIO.LG.AS[1,1]

meanu.BIO.LG.ADT<- MX.BIO.LG.ADT%*%UTr.D.ADT

meanu.BIO.LG.ADT[1,1]<-(1-dUBiopsy-dUPCa-dUADT)*MX.BIO.LG.ADT[1,1]

meanu.BIO.LG.MD<- MX.BIO.LG.MD%*%UTr.LG.MD

meanu.BIO.LG.MD[1,1]<-1*MX.BIO.LG.MD[1,1]

meanu.BIO.NoPCa.P<- MX.BIO.NoPCa.P%*%UTr.D.NoPCa

meanu.BIO.NoPCa.P[1,1]<-(1-dUBiopsy)*MX.BIO.NoPCa.P[1,1]

meanu.BIO.NoPCa.N<- MX.BIO.NoPCa.N%*%UTr.D.NoPCa

meanu.BIO.NoPCa.N[1,1]<-MX.BIO.NoPCa.N[1,1]

meanu.BIO<-meanu.BIO.HG.RP+meanu.BIO.HG.RT+meanu.BIO.HG.ADT+meanu.BIO.HG.WW+meanu.BIO.HG.MD+meanu.BIO.LG.MD+

meanu.BIO.LG.RP+meanu.BIO.LG.RT+meanu.BIO.LG.ADT+meanu.BIO.LG.AS+meanu.BIO.NoPCa.P+meanu.BIO.NoPCa.N

#############################

DisU.BIO.popdata<- t(meanu.BIO) %*% cycle_rDiscount.U

v_tc_d <- c(DisC.SOC.popdata, DisC.BIO.popdata)

v_tu_d <- c(DisU.SOC.popdata, DisU.BIO.popdata)

df_ce <- data.frame(Strategy = Stra_names,

Cost = v_tc_d,

Effect = v_tu_d)

#NMB = v_nmb_d)

return(df_ce)

}

n.t<-18

state_names <- c("Long term", "Dead")

Stra_names<-c("Standard of Care", "Risk Assessment Tool")

n.states <- length(state_names)

p_mor_age_NoPCa1<-read.csv(file = 'age-mort.CSV')

p_mor_age_NoPCa<-p_mor_age_NoPCa1[,'p']

p_mor_age_HG1<-read.csv(file = 'age-mort-HG.CSV')

p_mor_age_HG<-p_mor_age_HG1[,'p']

p_mor_age_LG1<-read.csv(file = 'age-mort-LG.CSV')

p_mor_age_LG<-p_mor_age_LG1[,'p']

set.seed(1234)

file_path <- "Inputs.xlsx"

Param_data <- read_xlsx(file_path)

variable_names <- Param_data$Name

variable_values <- Param_data$Value

for (i in 1:length(variable_names)) {

assign(variable_names[i], variable_values[i])

}

p.PCa<-p.PCa

CBiomarker<-CBiomarker

CDead<-CDead

Bio.Spec.NoPca<-Bio.Spec.NoPca

Bio.Sens.HG<-Bio.Sens.HG

Bio.Spec.LG<-Bio.Spec.LG

n_sim<-1

df1_c <- as.data.frame(matrix(0,

nrow = 10000,

ncol = 2))

colnames(df1_c) <- Stra_names

df1_e <- as.data.frame(matrix(0,

nrow = 10000,

ncol = 2))

colnames(df1_e) <- Stra_names

####################

for(i in 1:10000){

df_out_temp <- df_out_ce(18)

df1_c[i, ] <- df_out_temp$Cost

df1_e[i, ] <- df_out_temp$Effect

# Display simulation progress

if(i/(n_sim/10) == round(i/(n_sim/10), 0)) {

cat('\r', paste(i/n_sim * 100, "% done", sep = " "))

}

}

**References**

1. Rendon RA, Mason RJ, Marzouk K, et al. Canadian Urological Association recommendations on prostate cancer screening and early diagnosis. *Can Urol Assoc J*. 2017;11(10):298-309. doi:10.5489/cuaj.4888

2. Schröder FH, Hugosson J, Roobol MJ, et al. Screening and prostate-cancer mortality in a randomized European study. *N Engl J Med*. 2009;360(13):1320-1328. doi:10.1056/NEJMoa0810084

3. Dijkstra S, Mulders PFA, Schalken JA. Clinical use of novel urine and blood based prostate cancer biomarkers: a review. *Clin Biochem*. 2014;47(10-11):889-896. doi:10.1016/j.clinbiochem.2013.10.023

4. Polascik TJ, Oesterling JE, Partin AW. Prostate specific antigen: a decade of discovery--what we have learned and where we are going. *J Urol*. 1999;162(2):293-306. doi:10.1016/s0022-5347(05)68543-6

5. Thompson IM, Ankerst DP, Chi C, et al. Operating characteristics of prostate-specific antigen in men with an initial PSA level of 3.0 ng/ml or lower. *JAMA*. 2005;294(1):66-70. doi:10.1001/jama.294.1.66

6. Ministry of Health. Prostate cancer part 1: Diagnosis and referral in primary care - Province of British Columbia. Published June 28, 2022. Accessed June 28, 2022. https://www2.gov.bc.ca/gov/content/health/practitioner-professional-resources/bc-guidelines/prostate-cancer-part-1

7. Barry MJ. Clinical practice. Prostate-specific-antigen testing for early diagnosis of prostate cancer. *N Engl J Med*. 2001;344(18):1373-1377. doi:10.1056/NEJM200105033441806

8. Tawfik A. Prostate-specific antigen (PSA)–based population screening for prostate cancer: An economic analysis. *Ont Health Technol Assess Ser*. 2015;15(11):1-37.

9. Wolf AMD, Wender RC, Etzioni RB, et al. American Cancer Society guideline for the early detection of prostate cancer: Update 2010. *CA Cancer J Clin*. 2010;60(2):70-98. doi:10.3322/caac.20066

10. Raaijmakers R, Kirkels WJ, Roobol MJ, Wildhagen MF, Schrder FH. Complication rates and risk factors of 5802 transrectal ultrasound-guided sextant biopsies of the prostate within a population-based screening program. *Urology*. 2002;60(5):826-830. doi:10.1016/S0090-4295(02)01958-1

11. Fenton JJ, Weyrich MS, Durbin S, Liu Y, Bang H, Melnikow J. Prostate-specific antigen–based screening for prostate cancer: evidence report and systematic review for the US Preventive Services Task Force. *JAMA*. 2018;319(18):1914-1931. doi:10.1001/jama.2018.3712

12. Etzioni R, Penson DF, Legler JM, et al. Overdiagnosis due to prostate-specific antigen screening: lessons from U.S. prostate cancer incidence trends. *J Natl Cancer Inst*. 2002;94(13):981-990. doi:10.1093/jnci/94.13.981

13. Draisma G, Etzioni R, Tsodikov A, et al. Lead time and overdiagnosis in prostate-specific antigen screening: importance of methods and context. *J Natl Cancer Inst*. 2009;101(6):374-383. doi:10.1093/jnci/djp001

14. Yoshida K, Honda M, Sumi S, Arai K, Suzuki S, Kitahara S. Levels of free prostate-specific antigen (PSA) can be selectively measured by heat treatment of serum: free/total-PSA ratios improve detection of prostate carcinoma - PubMed. *Clin Chim Acta*. 1999;280(1-2):195-203. doi:10.1016/S0009-8981(98)00189-2

15. Liu J, Dong B, Qu W, et al. Using clinical parameters to predict prostate cancer and reduce the unnecessary biopsy among patients with PSA in the gray zone. *Sci Rep*. 2020;10(1):5157. doi:10.1038/s41598-020-62015-w

16. Park BK, Park JW, Park SY, et al. Prospective evaluation of 3-T MRI performed before initial transrectal ultrasound-guided prostate biopsy in patients with high prostate-specific antigen and no previous biopsy. *AJR Am J Roentgenol*. 2011;197(5):W876-881. doi:10.2214/AJR.11.6829

17. Haffner J, Lemaitre L, Puech P, et al. Role of magnetic resonance imaging before initial biopsy: comparison of magnetic resonance imaging-targeted and systematic biopsy for significant prostate cancer detection. *BJU Int*. 2011;108(8 Pt 2):E171-178. doi:10.1111/j.1464-410X.2011.10112.x

18. Kasivisvanathan V, Rannikko AS, Borghi M, et al. MRI-targeted or standard biopsy for prostate-cancer diagnosis. *N Engl J Med*. 2018;378(19):1767-1777. doi:10.1056/NEJMoa1801993

19. Dijkstra S, Govers TM, Hendriks RJ, et al. Cost-effectiveness of a new urinary biomarker-based risk score compared to standard of care in prostate cancer diagnostics – a decision analytical model. *BJU Int*. 2017;120(5):659-665. doi:10.1111/bju.13861

20. Govers TM, Caba L, Resnick MJ. Cost-effectiveness of urinary biomarker panel in prostate cancer risk assessment. *J Urol*. 2018;200(6):1221-1226. doi:10.1016/j.juro.2018.07.034

21. Sathianathen NJ, Kuntz KM, Alarid-Escudero F, et al. Incorporating biomarkers into the primary prostate biopsy setting: A cost-effectiveness analysis. *J Urol*. 2018;200(6):1215-1220. doi:10.1016/j.juro.2018.06.016

22. Sari Motlagh R, Yanagisawa T, Kawada T, et al. Accuracy of SelectMDx compared to mpMRI in the diagnosis of prostate cancer: a systematic review and diagnostic meta-analysis. *Prostate Cancer Prostatic Dis*. 2022;25(2):187-198. doi:10.1038/s41391-022-00538-1

23. Venderink, W., Govers, T.M., Rooij, M. de, Futterer, J.J., Sedelaar, J.P.M. Cost-Effectiveness Comparison of Imaging-Guided Prostate Biopsy Techniques: Systematic Transrectal Ultrasound, Direct In-Bore MRI, and Image Fusion. *Am J Roentgenol*. 2017;208:1058-1063. doi:10.2214/ajr.16.17322

24. Faria R, Soares MO, Spackman E, et al. Optimising the Diagnosis of Prostate Cancer in the Era of Multiparametric Magnetic Resonance Imaging: A Cost-effectiveness Analysis Based on the Prostate MR Imaging Study (PROMIS). *Eur Urol*. 2018;73(1):23-30. doi:10.1016/j.eururo.2017.08.018

25. Cerantola Y, Dragomir A, Tanguay S, Bladou F, Aprikian A, Kassouf W. Cost-effectiveness of multiparametric magnetic resonance imaging and targeted biopsy in diagnosing prostate cancer. *Urol Oncol*. 2016;34(3):119.e1-9. doi:10.1016/j.urolonc.2015.09.010

26. Keeney E, Thom H, Turner E, Martin RM, Morley J, Sanghera S. Systematic review of cost-effectiveness models in prostate cancer: Exploring new developments in testing and diagnosis. *Value Health*. 2022;25(1):133-146. doi:10.1016/j.jval.2021.07.002

27. Lee S. Finding prostate cancer early. Canadian Cancer Society. Published September 2022. Accessed March 30, 2023. https://cancer.ca/en/cancer-information/cancer-types/prostate/finding-cancer-early

28. Govers TM, Hessels D, Vlaeminck-Guillem V, et al. Cost-effectiveness of SelectMDx for prostate cancer in four European countries: a comparative modeling study. *Prostate Cancer Prostatic Dis*. 2019;22(1):101-109. doi:10.1038/s41391-018-0076-3

29. Govers TM, Caba L, Resnick MJ. Cost-Effectiveness of Urinary Biomarker Panel in Prostate Cancer Risk Assessment. *J Urol*. 2018;200(6):1221-1226. doi:10.1016/j.juro.2018.07.034

30. Bill-Axelson A, Holmberg L, Garmo H, et al. Radical prostatectomy or watchful waiting in early prostate cancer. *N Engl J Med*. 2014;370(10):932-942. doi:10.1056/nejmoa1311593

31. BC Cancer Registry Data (2021). BC Cancer [publisher]. Data Extract. BC Cancer (2021). Published September 28, 2021. Accessed September 28, 2021. http://www.bccancer.bc.ca/health-professionals/professional-resources/bc-cancer-registry/request-registry-data

32. BC Cancer Registry Data (2021). V2. Population Data BC [publisher]. Data Extract. BC Cancer (2021). Published September 28, 2021. Accessed September 28, 2021. https://www.popdata.bc.ca/data

33. British Columbia Ministry of Health [creator] (2021). Medical Services Plan (MSP) Payment Information File. V2. Population Data BC [publisher]. Data Extract. MOH (2021). Published September 28, 2021. Accessed September 28, 2021. http://www.popdata.bc.ca/data

34. BC Ministry of Health [creator] (2021). PharmaNet. V2. BC Ministry of Health [publisher]. Data Extract. Data Stewardship Committee (2021). Published September 28, 2021. Accessed September 28, 2021. https://www.popdata.bc.ca/data

35. Canadian Institute for Health Information [creator] (2021). Discharge Abstract Database (Hospital Separations). V2. Population Data BC [publisher]. Data Extract. MOH (2021). Published September 28, 2021. Accessed September 28, 2021. https://www.popdata.bc.ca/data

36. Canadian Institute for Health Information [creator] (2021). National Ambulatory Care Reporting System. V2. Population Data BC [publisher]. Data Extract. MOH (2021). Published September 28, 2021. Accessed September 28, 2021. https://www.popdata.bc.ca/data

37. British Columbia Ministry of Health [creator] (2021). Vital Events Deaths. Population Data BC [publisher]. Data Extract. MOH (2021). Published September 28, 2021. Accessed September 28, 2021. https://www.popdata.bc.ca/data

38. British Columbia Ministry of Health [creator] (2021). Consolidation File (MSP Registration & Premium Billing). V2. Population Data BC [publisher]. Data Extract. MOH (2021). Published September 28, 2021. Accessed September 28, 2021. http://www.popdata.bc.ca/data

39. Zhang W, Guh DP, Mohammadi T, et al. Health Care Costs Attributable to Prostate Cancer in British Columbia, Canada: A Population-Based Cohort Study. *Curr Oncol*. 2023;30(3):3176-3188. doi:10.3390/curroncol30030240

40. Chang EK, Gadzinski AJ, Nyame YA. Blood and urine biomarkers in prostate cancer: Are we ready for reflex testing in men with an elevated prostate-specific antigen? *Asian J Urol*. 2021;8(4):343-353. doi:10.1016/j.ajur.2021.06.003

41. del Pino-Sedeño T, Infante-Ventura D, de Armas Castellano A, et al. Molecular Biomarkers for the Detection of Clinically Significant Prostate Cancer: A Systematic Review and Meta-analysis. *Eur Urol Open Sci*. 2022;46:105-127. doi:10.1016/j.euros.2022.10.017

42. Loeb S, Lilja H, Vickers A. Beyond PSA: Utilizing Novel Strategies to Screen Men for Prostate Cancer. *Curr Opin Urol*. 2016;26(5):459-465. doi:10.1097/MOU.0000000000000316

43. Van Neste L, Hendriks RJ, Dijkstra S, et al. Detection of High-grade Prostate Cancer Using a Urinary Molecular Biomarker–Based Risk Score. *Eur Urol*. 2016;70(5):740-748. doi:10.1016/j.eururo.2016.04.012

44. Hendriks RJ, van der Leest MMG, Israël B, et al. Clinical use of the SelectMDx urinary-biomarker test with or without mpMRI in prostate cancer diagnosis: a prospective, multicenter study in biopsy-naïve men. *Prostate Cancer Prostatic Dis*. 2021;24(4):1110-1119. doi:10.1038/s41391-021-00367-8

45. Russo GI, Regis F, Castelli T, et al. A systematic review and meta-analysis of the diagnostic accuracy of Prostate Health Index and 4-kallikrein panel score in predicting overall and high-grade prostate cancer. *Clin Genitourin Cancer*. 2017;15(4):429-439.e1. doi:10.1016/j.clgc.2016.12.022

46. Duffy MJ. Biomarkers for prostate cancer: prostate-specific antigen and beyond. *Clin Chem Lab Med CCLM*. 2020;58(3):326-339. doi:10.1515/cclm-2019-0693

47. Braun K, Sjoberg DD, Vickers AJ, Lilja H, Bjartell AS. A four-kallikrein panel predicts high-grade cancer on biopsy: Independent validation in a community cohort. *Eur Urol*. 2016;69(3):505-511. doi:10.1016/j.eururo.2015.04.028

48. Heijnsdijk EAM, Wever EM, Auvinen A, et al. Quality-of-life effects of prostate-specific antigen screening. *N Engl J Med*. 2012;367(7):595-605. doi:10.1056/NEJMoa1201637

49. Venderink, W., Govers, T.M., Rooij, M. de, Futterer, J.J., Sedelaar, J.P.M. Cost-effectiveness comparison of imaging-guided prostate biopsy techniques: Systematic transrectal ultrasound, direct in-bore MRI, and image fusion. *Am J Roentgenol*. 2017;208:1058-1063. doi:10.2214/ajr.16.17322

50. Karlsson AA, Hao S, Jauhiainen A, et al. The cost-effectiveness of prostate cancer screening using the Stockholm3 test. *PLOS ONE*. 2021;16(2):e0246674. doi:10.1371/journal.pone.0246674

51. Heijnsdijk EAM, Denham D, de Koning HJ. The cost-effectiveness of prostate cancer detection with the use of Prostate Health Index. *Value Health*. 2016;19(2):153-157. doi:10.1016/j.jval.2015.12.002

52. Guidelines for the Economic Evaluation of Health Technologies: Canada | CADTH. Accessed August 2, 2022. https://www.cadth.ca/guidelines-economic-evaluation-health-technologies-canada-0

53. Pahwa S, Schiltz NK, Ponsky LE, Lu Z, Griswold MA, Gulani V. Cost-effectiveness of MR imaging-guided strategies for detection of prostate cancer in biopsy-naive men. *Radiology*. 2017;285(1):157-166. doi:10.1148/radiol.2017162181

54. Faria R, Soares MO, Spackman E, et al. Optimising the diagnosis of prostate cancer in the era of multiparametric magnetic resonance imaging: A cost-effectiveness analysis based on the prostate MR imaging study (PROMIS). *Eur Urol*. 2018;73(1):23-30. doi:10.1016/j.eururo.2017.08.018

55. de Rooij M, Crienen S, Witjes JA, Barentsz JO, Rovers MM, Grutters JPC. Cost-effectiveness of magnetic resonance (MR) imaging and MR-guided targeted biopsy versus systematic transrectal ultrasound-guided biopsy in diagnosing prostate cancer: a modelling study from a health care perspective. *Eur Urol*. 2014;66(3):430-436. doi:10.1016/j.eururo.2013.12.012

56. Bouttell J, Teoh J, Chiu PK, et al. Economic evaluation of the introduction of the Prostate Health Index as a rule-out test to avoid unnecessary biopsies in men with prostate specific antigen levels of 4-10 in Hong Kong. *PLOS ONE*. 2019;14(4):e0215279. doi:10.1371/journal.pone.0215279

57. Inoue LYT, Etzioni R, Morrell C, Müller P. Modeling Disease Progression with Longitudinal Markers. *J Am Stat Assoc*. 2008;103(481):259-270. doi:10.1198/016214507000000356

58. Pataky R, Gulati R, Etzioni R, et al. Is prostate cancer screening cost-effective? A microsimulation model of prostate-specific antigen-based screening for British Columbia, Canada. *Int J Cancer*. 2014;135(4):939-947. doi:10.1002/ijc.28732

59. Etzioni R, Tsodikov A, Mariotto A, et al. Quantifying the role of PSA screening in the US prostate cancer mortality decline. *Cancer Causes Control CCC*. 2008;19(2):175-181. doi:10.1007/s10552-007-9083-8

60. Nichol MB, Wu J, Huang J, Denham D, Frencher SK, Jacobsen SJ. Cost-effectiveness of Prostate Health Index for prostate cancer detection. *BJU Int*. 2012;110(3):353-362. doi:10.1111/j.1464-410X.2011.10751.x

61. Teoh JYC, Leung CH, Wang MH, et al. The cost-effectiveness of prostate health index for prostate cancer detection in Chinese men. *Prostate Cancer Prostatic Dis*. 2020;23(4):615-621. doi:10.1038/s41391-020-0243-1

62. Lee J, Yang SW, Jin L, et al. Is PSA density of the peripheral zone as a useful predictor for prostate cancer in patients with gray zone PSA levels? *BMC Cancer*. 2021;21(1):472. doi:10.1186/s12885-021-08216-6

63. Government of Canada SC. Consumer Price Index, annual average, not seasonally adjusted. Published June 19, 2007. Accessed September 1, 2023. https://www150.statcan.gc.ca/t1/tbl1/en/tv.action?pid=1810000501

64. Quan H, Sundararajan V, Halfon P, et al. Coding algorithms for defining comorbidities in ICD-9-CM and ICD-10 administrative data. *Med Care*. 2005;43(11):1130-1139. doi:10.1097/01.mlr.0000182534.19832.83

65. Heijnsdijk EAM, Denham D, de Koning HJ. The Cost-Effectiveness of Prostate Cancer Detection with the Use of Prostate Health Index. *Value Health*. 2016;19(2):153-157. doi:10.1016/j.jval.2015.12.002

66. Stewart ST, Lenert L, Bhatnagar V, Kaplan RM. Utilities for Prostate Cancer Health States in Men Aged 60 and Older. *Med Care*. 2005;43(4):347-355.
